# The genetic history of Greenlandic-European contact

**DOI:** 10.1016/j.cub.2021.02.041

**Published:** 2021-03-11

**Authors:** Ryan K. Waples, Aviaja L. Hauptmann, Inge Seiding, Emil Jørsboe, Marit E. Jørgensen, Niels Grarup, Mette K. Andersen, Christina V.L. Larsen, Peter Bjerregaard, Garrett Hellenthal, Torben Hansen, Anders Albrechtsen, Ida Moltke

**Affiliations:** 1 Section for Computational and RNA Biology, Department of Biology, University of Copenhagen, Copenhagen, Denmark; 2 Ilisimatusarfik - The University of Greenland, Nuuk, Greenland; 3 Nunatta Katersugaasivia Allagaateqarfialu - Greenland National Museum and Archives, Nuuk, Greenland; 4 Novo Nordisk Foundation Center for Basic Metabolic Research, Faculty of Health and Medical Sciences, University of Copenhagen, Copenhagen, Denmark; 5 National Institute of Public Health, University of Southern Denmark, Copenhagen, Denmark; 6 Greenland Centre for Health Research, University of Greenland, Nuuk, Greenland; 7 Steno Diabetes Center Copenhagen, Gentofte, Denmark; 8 UCL Genetics Institute (UGI), Department of Genetics, Evolution and Environment, UCL, London, UK; 9 Faculty of Health Sciences, University of Southern Denmark, Odense, Denmark; 10 These authors contributed equally; 11 Lead contact

## Abstract

The Inuit ancestors of the Greenlandic people arrived in Greenland close to 1,000 years ago.^1^ Since then, Europeans from many different countries have been present in Greenland. Consequently, the present-day Greenlandic population has ~25% of its genetic ancestry from Europe.^2^ In this study, we investigated to what extent different European countries have contributed to this genetic ancestry. We combined dense SNP chip data from 3,972 Greenlanders and 8,275 Europeans from 14 countries and inferred the ancestry contribution from each of these 14 countries using haplotype-based methods. Due to the rapid increase in population size in Greenland over the past ~100 years, we hypothesized that earlier European interactions, such as pre-colonial Dutch whalers and early German and Danish-Norwegian missionaries, as well as the later Danish colonists and post-colonial immigrants, all contributed European genetic ancestry. However, we found that the European ancestry is almost entirely Danish and that a substantial fraction is from admixture that took place within the last few generations.

## In brief

The Greenlandic Inuit have had extensive historical contact with Europeans, and the present-day Greenlandic population has substantial amounts of European ancestry. Waples et al. use genetic data to investigate the origin of this ancestry. They show that much of it is Danish and find little evidence of it being from pre-colonial European contact.

## RESULTS

### Background

**Figure 1.**
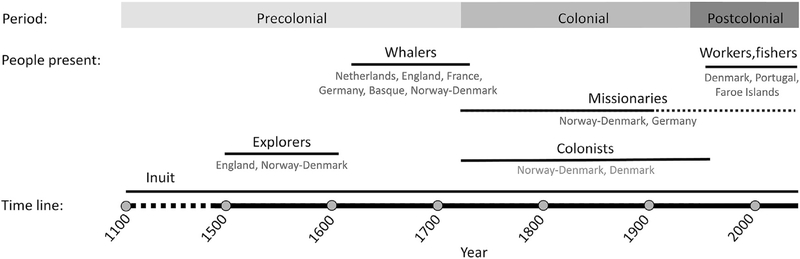
Timeline of significant Greenlandic-European contact The timeline covers the time since the arrival of the Inuit in Greenland. The presence of different groups of people are shown by horizontal bars. Times are approximate. The most relevant European countries are listed under each group.

The Greenlanders are mainly descendants of the Inuit of the Thule culture^3^ that entered Northern Greenland from Canada around the 12^th^ century.^1,4^ At that time, the Norse had lived in the southern part of the island since 985 CE; they stayed in Greenland until approximately 1450 CE. Previous genetic research has not provided support for gene flow between the Norse and Inuit.^2^ However, since the 16^th^ century, thousands of Europeans from various countries either visited or moved to Greenland, and there has been substantial gene flow from Europe into the Greenlandic population.^2,5–7^ This European contact with Greenland can be divided into three time periods: pre-colonial; colonial; and post-colonial (Figure 1). Pre-colonial contact was initially limited to exploration and trade, such as when a search for the Northwest Passage led English explorers to Greenland in the 1500s.^8^ From the early 18^th^ century, European whaling efforts off the west coast of Greenland brought whalers into contact with the Greenlandic Inuit. Initially, it was the Dutch who dominated European whaling, but in the latter half of the century, the whalers were also German, primarily Frisian, British, and Danish-Norwegian.^8,9^ In 1721, the arrival of the Danish-Norwegian missionary priest Hans Egede marked the beginning of the colonial period, leading to a new and more permanent type of contact between Greenlandic Inuit and Europeans, although whaling was still a primary draw, with, e.g., 107 Dutch ships that year. In addition to Danish-Norwegian missionaries, the German Moravian Brethren established religious missions in the period 1733–1900, located in Nuuk and several other locations.^8^ In 1751, Denmark-Norway expanded colonial activities and claimed a monopoly on trade,^8^ and since then, the primary contact between Greenlanders and Europeans has been with the Kingdom of Denmark. Denmark-Norway remained a conglomerate state until 1814,^10^ after which Greenland became an exclusively Danish colony, and in 1953, it became an equal part of the Kingdom of Denmark. The post-colonial period has seen a significant influx of mainly Danish workers but also seasonal fishers, primarily from Portugal and the Faroe Islands.^8,11^

Although there was a systematic documentation of marriages between Inuit and Scandinavians from the 1740s,^8,10,12^ the degree of admixture prior to the colonial period is largely unknown. However, the extensive whaling and trading activities of the Dutch have led to a common belief in Greenland that admixture with Dutch whalers was relatively common.^13^ And notably, any European contribution to the Greenlandic gene pool prior to the 20^th^ century could have an outsized impact compared to more recent admixture, because the population of Greenland has recently greatly expanded, from less than 6,000 in 1789 to more than 55,000 today.^14^ Thus, prior to performing this study, we hypothesized that especially Denmark but also the Netherlands, Germany, and Norway all made non-negligible ancestry contributions. Here, we investigated this hypothesis by analyzing dense SNP array data from 3,972 Greenlanders from 15 different locations (Figure S1).

### Inference of European admixture sources

Using the genetic data from the Greenlanders, we inferred admixture proportions (Figure S2; see also Data S1) and identified related individuals. Based on the results, we obtained a set of unrelated admixed Greenlanders with both Inuit and European ancestry (n = 1,582) and a set of 181 unrelated, unadmixed Greenlanders with only Inuit ancestry. Genetic data from these individuals were then combined with SNP array data from 8,275 individuals from 14 different European countries, including Denmark, Norway, Sweden, Germany, the UK, and the Netherlands (Figure S1). After quality control and merging, we were left with a combined dataset with 135,702 SNPs, 1,582 admixed Greenlanders, and 8,456 reference individuals. Next, we applied the program ChromoPainter^15^ to this dataset to reconstruct (“paint”) the genomes of the admixed Greenlandic individuals as mixtures of the haplotypes in the reference individuals (Table S1). The main outcome of this analysis was an estimate for each admixed Greenlander (and each reference individual) of over how much of their genome they are most closely related ancestrally to each of the reference individuals. These estimates were summarized in a so-called coancestry matrix (Figure S3).

We then estimated the genetic contribution from the unadmixed Greenlandic Inuit and each of the 14 different European countries to the ancestry of the admixed individuals in Greenland by applying the program SOURCEFIND^16^ to a summary of the output from ChromoPainter. SOURCEFIND is a Markov chain Monte Carlo (MCMC) method that produces statistical samples from a posterior distribution of the ancestry contributions for a group of target individuals. In this case, the target individuals were the admixed Greenlanders and the possible ancestry sources were the reference countries. These statistical samples can then be summarized in different ways that are informative about the ancestral contribution of each of the reference countries. We applied this method to each admixed Greenlander separately to obtain individual-level resolution but also tried to analyze all the admixed Greenlanders jointly in one group in an attempt to reduce noise in our ancestry estimates.

**Figure 2.**
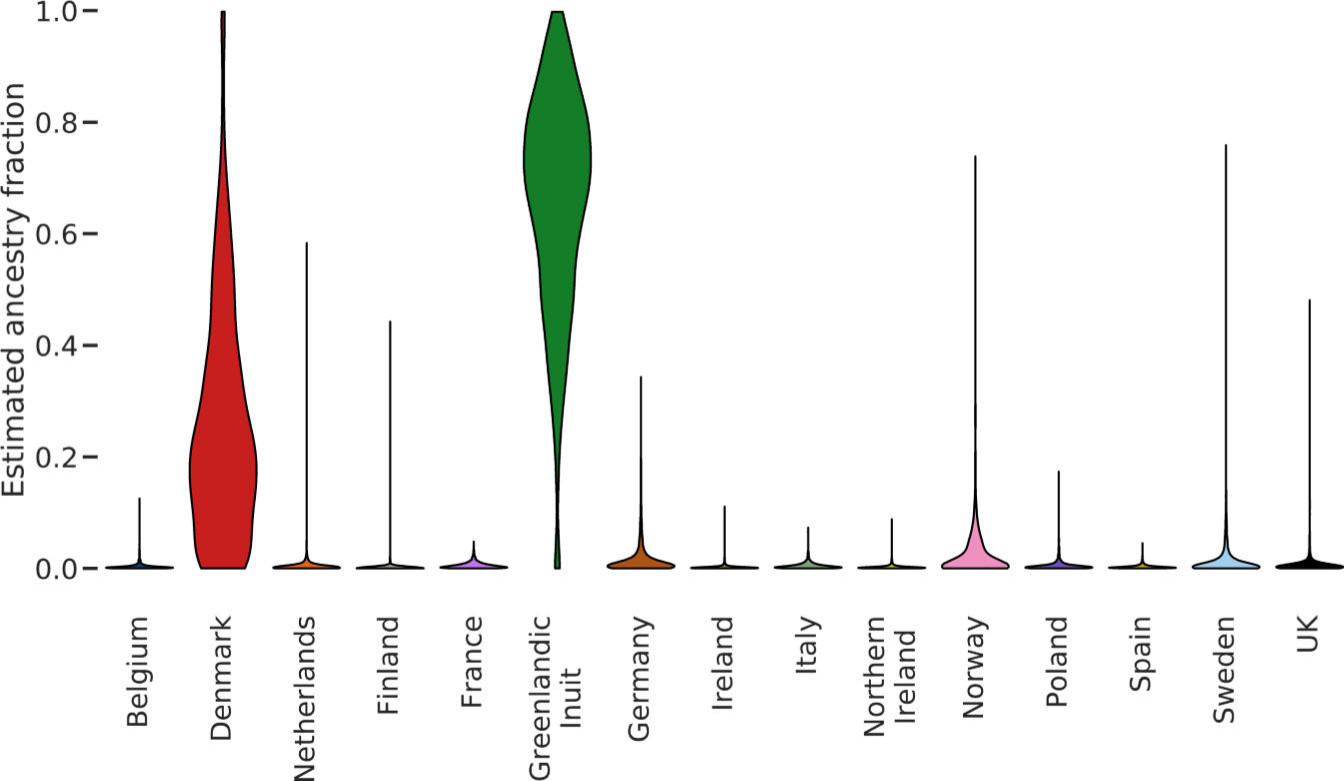
Violin plot of per-country ancestry estimates Results shown are produced by SOURCEFIND from the analysis where inference was performed on each admixed individual separately. Each source country has a violin showing the distribution of the estimated mean ancestry fraction from that country, across all admixed individuals. Each admixed individual appears in the distribution for each country. Violins are scaled to all have the same max width.

When performing inference on each admixed individual separately, we first summarized the SOURCEFIND results using the posterior mean of the ancestry proportion from each country per individual to get a simple overview (Figure 2). Based on this, we estimated the 1,582 admixed Greenlanders to have an average of 65.6% Greenlandic Inuit ancestry and 34.4% European ancestry, with the far majority of the latter being Danish. To obtain a more detailed picture of the SOURCEFIND results, we also counted the number of individuals that were assigned at least 5% ancestry from any country with high probability (posterior probability > 0.99). When doing so, we found 1,100 of the 1,582 admixed Greenlandic individuals (69.5%) were assigned at least 5% Danish ancestry, the most of any European country (Table 1). The only other European countries found to contribute more than 5% ancestry to five or more individuals are all Nordic countries: Norway with 98 (6.2%); Sweden with 20 (1.3%); and Finland with 5 (0.3%). A few other countries were inferred to contribute more than 5% ancestry for 1–5 individuals: UK (0.2%); Ireland (0.2%); Poland (0.1%); Germany (0.1%); and the Netherlands (0.1%). The same overall pattern is observed with a lower 1% ancestry threshold (Table 1), with different posterior probability thresholds or a different prior (Table S2). Only four individuals with more than 20% European ancestry were inferred to have ancestry from countries other than Denmark or Norway (Table 1).

We obtained similar results in the group-based analysis, with an estimated Inuit ancestry fraction of 65.6% and European fraction of 34.4% (95% credible interval = 33.5%–36.0%) (Table 1). Please note these results pertain to the admixed Greenlandic individuals and do not reflect the Greenlandic population as a whole, which is estimated to have approximately 25% European ancestry.^2^ The Danish ancestry fraction among admixed Greenlanders was 31%, with no other European country contributing more than 1%. This translates to Denmark making up 91% of the total estimated European ancestry, with the only other country contributing more than 1% of the European ancestry being Norway at 2.1% (Table 1). Notably, we performed a range of additional analyses to ensure the validity of these results (Data S2).

### Investigating European admixture in the last few generations

**Figure 3.**
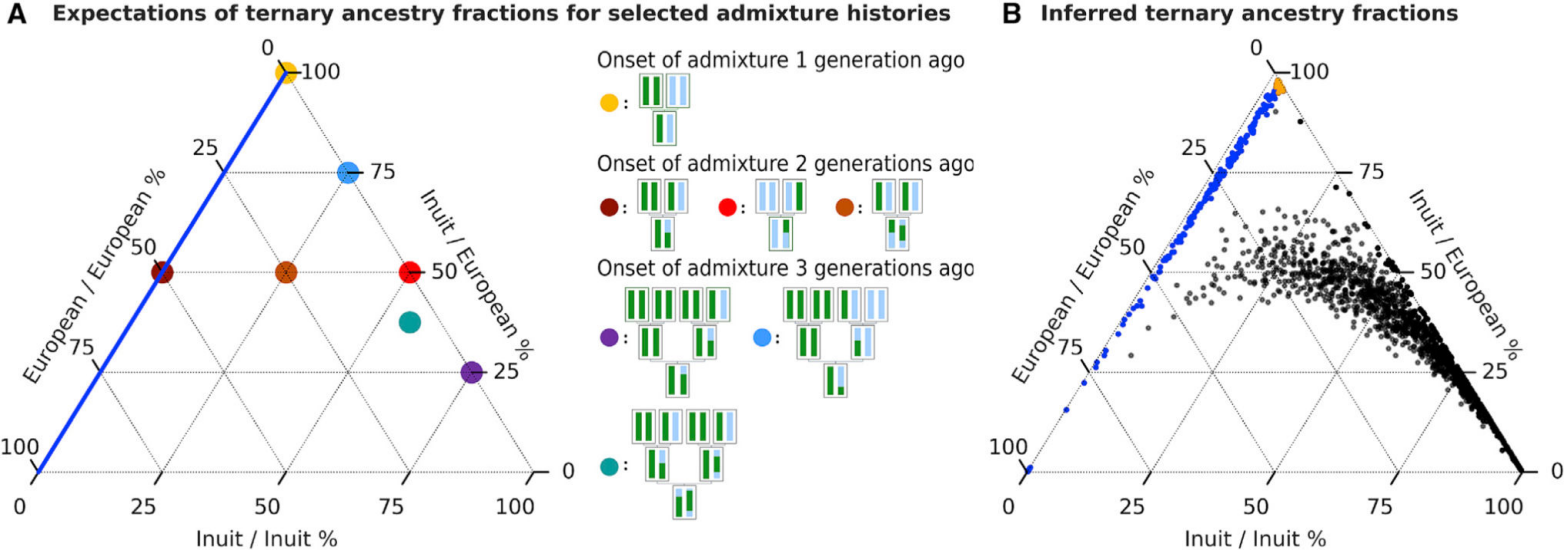
Ternary ancestry fractions, i.e., the fraction of the genome where (1) both alleles have Inuit ancestry, (2) both alleles have European ancestry, or (3) one allele has Inuit ancestry and one has European ancestry The three corners of the plots represent genomes with all loci having two European alleles (bottom left), two Inuit alleles (bottom right), or one Inuit and one European alleles (top). (A) Expected ternary ancestry fractions. Colored dots show the expected ternary fractions for individuals with 7 selected admixture histories illustrated in the legend by pedigrees, where green indicates Inuit ancestry and light blue indicates European ancestry. The admixture histories include Greenlanders with admixture from one European parent (yellow); one European grandparent (dark brown); two European grandparents, one on each parental side (red); three European grandparents (light brown); one European great-grandparent (blue), two European great-grandparents, one on each parental side (blue-green); and, finally, three European great-grandparents, all on the same parental side (purple). The left axis in blue indicates fractions that are expected for individuals with at least one European parent because it has no sites with two Inuit alleles. (B) Inferred ternary ancestry fractions. Colored dots show the inferred ternary ancestry fractions for each of the 1,582 admixed Greenlanders. The colors convey the way we have categorized the individuals: individuals inferred to have one Greenlandic parent and one European parent are yellow; the remaining individuals inferred to have a European parent shown in blue; and other individuals are shown in black. See also Table S3.

To further characterize the history of European admixture in Greenland, we performed an analysis to investigate the timing of admixture in Greenland. Specifically, we inferred local ancestry, Inuit or European, along the genome of each admixed Greenlander to estimate the proportions of the genome where each admixed individual has (1) inherited both alleles from Inuit ancestors, (2) inherited both alleles from European ancestors, or (3) inherited one allele from an Inuit ancestor and one allele from a European ancestor. These fractions are informative about the time of admixture because individuals with different admixture histories have different expected ternary fractions (see Figure 3A for some examples). We chose to estimate “ternary ancestry fractions” instead of using standard methods for timing of admixture based on admixture tract lengths (e.g., Pool and Nielsen^17^ and Gravel^18^), because the number of phasing switch errors was large relative to the recombination rate since admixture, which we feared would markedly affect the timing estimates. In contrast, the ternary ancestry fractions are robust to phasing switch errors.

Among the 1,582 admixed Greenlanders, 250 have ternary fractions that are consistent with having at least one fully European ancestry parent (Figure 3B, blue and yellow dots). Of these, 27 have two European alleles at nearly every genomic position (yellow dots on Figure 3B), suggesting they have two European parents. Together, these 277 (223 + 2 × 27) European ancestry parents account for >8% of the ancestors of the admixed individuals (277/[2 × 1,582]) and for almost 25% of the total European ancestry in Greenland. The ternary ancestry fractions of the remaining individuals are largely consistent with second and third generation admixture with Europeans, as shown in Figure 3A (dots near the right axis). However, it is important to emphasize that, due to variance in recombination and nonrandom mating, these fractions could also be the result of older admixture.

Among the group of admixed Greenlanders with at least one European-ancestry parent, Denmark was by far the largest European ancestry source, making up 98.4% of the European ancestry, with no other country contributing more than 1% (Table S3). In contrast, the group of Greenlanders without a European parent, i.e., a group for which the admixture must have taken place less recently than for the group with at least one European parent, was inferred to have contributions from Norway (3.8%), Germany (2.1%), and Sweden (1.6%), with Denmark constituting 85.7%.

## DISCUSSION

Before discussing the results in a historical perspective, we should consider how the study design imposes limitations in the conclusions that can be drawn from the analysis. Briefly, we do not believe that the current study imposes major limitations that pertain to the conclusions presented here; for further details and discussion on this topic, please see Data S2.

The genetic analyses suggest that the European ancestry of the present-day Greenlanders is predominantly Danish and the result of very recent gene flow. This indicates that European activities prior to colonization did not have a significant impact on the current genetic composition of the population in Greenland, in contrast both to common beliefs in Greenland^13^ and our own initial hypothesis.

The lack of genetic ancestry originating from the early exploration activities by the British is perhaps the least surprising, because these activities only involved a few ships. Similarly, the relatively small amount of ancestry originating from German Moravian missionaries, who stayed in Greenland for about 170 years until 1900, may be explained by the restrictions that the Moravian Brethren put on intermarriage with the Greenlandic population.^19^

However, the lack of ancestry from whaling countries, especially the Netherlands, is surprising given the common beliefs in Greenland as well as the historical records suggesting a high number of Dutch ships around Greenland’s coasts for a substantial period of time.^8,9^ This result may be explained by a number of factors. First, early European whalers often did not spend the winter in Greenland.^13^Second, Dutch, English, and other European whaling activities were reduced by the economic monopoly imposed in 1751 by Denmark-Norway. Finally, it has been postulated that first contact with Europeans was followed by severe epidemics and that the interaction with the Dutch around Disko Island led to some of the first incidents of epidemics in the region.^13^ A well-documented example was a severe smallpox epidemic in Nuuk in the 1730s following the arrival of European ships.^13^ It is possible that these epidemics could have impacted early patterns of European ancestry and reduced the impact of early admixture.

Our results suggest that most of the European ancestry is from after colonization was initiated. This result is consistent with the fact that most of the Greenlandic individuals without any European ancestry live in the very north as well as the east coast of Greenland,^2^ because colonial activities were initiated later in the north (1909) and east (1894) than in the southwest (1721). Also, we found a higher fraction of Norwegian, Swedish, and German ancestry among the Greenlanders without at least one European-ancestry parent, which aligns well with the family registries from the colonial period. The large amount of inferred Danish ancestry, especially within the last generation, is consistent with historical records showing that the influx of Danes to Greenland in the post-colonial period since the 1950s marked a substantial increase in the European immigration rate.

Hence, taken together, although at first perhaps surprising, the results of this study seem consistent with recent demographic trends in Greenland and with historical records of European contact.

## STAR★METHODS

### RESOURCE AVAILABILITY

#### Lead contact

Further information and requests should be directed to and will be fulfilled by the Lead Contact, Ida Moltke (ida@binf.ku.dk).

#### Materials availability

This study did not generate new unique reagents.

#### Data and code availability

The accession number for the Greenlandic genotype data from the Multi-ethnic genotyping array (MEGA) reported in this paper is EGA: EGAS00001004933. The Greenlandic genotype data onthe Metabochip are available from the European Genome-phenome Archive (https://ega-archive.org) under the accession EGA: EGAS00001002641. The European reference datasets are also available at the European Genome-phenome Archive with accessions EGAD00000000120, EGAD00010000124, EGAD00010000288, and EGAD00010000632. The 1000 Genomes data are publicly available from the 1000 Genomes Project (https://www.internationalgenome.org/data).

### EXPERIMENTAL MODEL AND SUBJECT DETAILS

Study participants were Greenlandic individuals from two population surveys: the Inuit Health in Transition (IHIT, n = 3115) and a survey consisting of Greenlanders living in Greenland (B99, n = 1401), and Greenlanders living in Denmark (BBH, n = 547).^30,31^ The cohorts have participants from 15 different locations in Greenland from Qaanaaq in the northwest to Tasiilaq in the southeast, (Figure S1) as well as Greenlanders living in Denmark.

The participants gave oral and written consent to participate in the health surveys and subsequently they were mailed information about the population genetics analyses with an option to opt out at any time. The approval for population genetics analyses was given by the Commission for Scientific Research in Greenland (project 2014–08, 2014–098017).

To represent potential European source countries, we selected individuals from 14 different European countries (n = 14,385): The UK, Sweden, Germany, Norway, Italy, Finland, Belgium, the Netherlands (Dutch), France, Ireland, Denmark, Spain, Northern Ireland, and Poland.

### METHOD DETAILS

#### Greenlandic genotype data

All Greenlandic participants were genotyped on two SNP arrays: the CardioMetaboChip (196,224 SNPs)^2,22,32^ and the Multi-Ethnic Global Array (~1.5M SNPs).^33^ Data from these two SNP arrays were merged on the plus strand and 3972 individuals with genotypes from both SNP arrays and a missing rate below 0.02 were retained. From these we removed singletons, sites not on an autosome, as well as sites with a significant (p <1e-10) deviation from Hardy-Weinberg equilibrium in a test that accounts for admixture.^29^

#### European genotype data

The European SNP array data are from the Wellcome Trust Case Control Consortium (EGAD00000000120, EGAD00010000124, EGAD00010000288, EGAD00010000632),^20,21^ and were selected to represent a broad spectrum of potential European admixture sources in Greenland (Figure S1). The European datasets were lifted to hg19 and put on the plus strand, and sites with rates of missing data > 0.05 were removed prior to merging. We also excluded sites within the MHC region and within the HsInv0501 inversion on chromosome 8, as well as sites with more than two alleles. Finally, we limited the number of individuals from each European country to at most 1000 and confirmed that there were no related individuals within each European country.

#### 1000 Genomes Data

For the ADMIXTURE analyses and local ancestry analyses with RFmix (see below) we selected the Han Chinese in Beijing (CHB), Yoruba in Ibadan (YRI), and Utah residents with Northern and Western European Ancestry (CEU) population samples from the Thousand Genomes Project (1000G),^23^ for a total of 310 individuals. We used the phased genotypes from phase 3 aligned to GRCh37.

#### Merged Greenlandic and European reference data

For the haplotype-based analyses we worked on a dataset where the Greenlandic data and the European reference samples were merged. We kept all sites present in both datasets and excluded 52 sites with more than 2% missing data. The resulting merged dataset had 135,702 loci and 12,247 individuals with a total genotyping rate of 0.9995 and all loci with a minor allele count of at least 5.

The merged Greenlandic-European dataset was split by chromosome and phased without a reference panel using SHAPEIT^34^ (v2.r904) with default settings, using the HapMap phase II recombination map for hg19.

After merging and phasing, we removed close relatives among all Greenlandic individuals by retaining at most one individual from each pair of individuals with a coefficient of relatedness > 0.2. Then we split the remaining Greenlanders into two sets based the results of a K = 2 ADMIXTURE: 1) the un-admixed Greenlanders with >99% inferred Inuit ancestry, and 2) the admixed Greenlanders with > % inferred European ancestry, for additional details see Data S1. From the second set, we removed seventeen Greenlandic individuals estimated to have >5% African or >7% Asian ancestry in a K = 4 ADMIXTURE analyses including 1000 genomes samples from China (CHB), Nigeria (YRI), the US (CEU). These thresholds were selected to exclude individuals that differed markedly from the majority of other Greenlandic individuals (data not shown) and to be able to avoid having to include any Asian and African reference samples in our fine-scale analyses. We also excluded admixed Greenlandic individuals living in Denmark as these individuals may be more likely to have Danish ancestry than other European ancestries. This left us with a dataset consisting of 1582 not closely related Greenlanders with European admixture (admixed samples), 181 not closely related unadmixed Greenlanders (Inuit reference samples), and 8303 European reference samples.

Based on the results of a pilot ChromoPainter analysis, we subsequently excluded 28 of the European reference samples because they were significant outliers (z-score > 5), based on comparing their total chunk counts to the rest of the individuals from their country (not shown). An atypically high number of chunks can be indicative of low data quality. This resulted in a final set of 8275 European reference samples (Figure S1) and thus 8275+181 = 8456 reference samples in total and 1582 not closely related Greenlanders with European admixture. These data were used to infer ancestry contributions, for details of this analysis see the Quantification and statistical analysis section and Data S2.

#### Merged Greenlandic and 1000G data

To construct a dataset for the ADMIXTURE and local ancestry analyses, we merged the Greenlandic genotype data with data from 310 individuals from three 1000G populations: Han Chinese in Beijing (CHB), Yoruba in Ibadan (YRI), and Utah residents with Northern and Western European Ancestry (CEU). We subsequently removed 46 sites with a greater than 0.25 frequency difference in the CEU individuals compared to the European admixture component in the K = 2 analysis (see below), retaining 521,622 overlapping sites.

### QUANTIFICATION AND STATISTICAL ANALYSIS

#### ADMIXTURE analyses

We performed two different ADMIXTURE^25^ analyses to facilitate the generation of input data for our main analyses: 1) an unsupervised K = 2 ADMIXTURE analysis of all 3972 Greenlandic individuals assuming an Inuit and a European ancestry component, following a previous study^2^ and 2) a supervised K = 4 ADMIXTURE analysis of the Greenlandic individuals combined with individuals of European, Asian and African descent to investigate if there are any ancestry from Asian and African populations. We used the K = 2 analysis (Figure S2) to create two sets of Greenlandic individuals: “unadmixed” and “admixed” with > 99% or < 99% Inuit ancestry respectively. These unadmixed individuals were used as a reference for the Greenlandic Inuit ancestry component, while the admixed individuals were the subject of the main analyses; for a more detailed discussion, see Data S1.

Before the unsupervised K = 2 analysis, we applied a minor allele frequency (MAF) filter of 0.05 to the Greenlandic dataset described above (n = 3972), resulting in a dataset with 538,514 sites. For the supervised K = 4 admixture analysis, we selected the Han Chinese in Beijing (CHB), Yoruba in Ibadan (YRI), and Utah residents with Northern and Western European Ancestry (CEU) populations as proxies for Asian, African, and European ancestry, respectively.

For each analysis, we ran each ADMIXTURE (v1.3.0) ten times and selected the run with the maximum likelihood, checking convergence by ensuring multiple other runs within two log-likelihood units.

#### Relatedness estimation

To estimate relatedness coefficients for the Greenlandic individuals we used relateAdmix.^28^ This method accounts for admixture by estimating individual allele frequencies when estimating pairwise identity by descent (IBD) coefficients (*k*_1_, *k*_2_) based on genome-wide ancestry proportions for each individual. We used the K = 2 genotype data and ADMIXTURE estimates of these genome-wide ancestry proportions. To estimate relatedness for the Europeans we applied the IBD inference function (−genome) in PLINK2^26,35^ to the genotype data from all the Europeans.

#### Chromosome painting

We characterized the coancestry between Greenlanders and Europeans with the haplotype-based method ChromoPainter.^15^ This method is based on a Hidden Markov model (HMM) that statistically reconstructs (“paints”) a target haplotype as a mixture of a set of reference haplotypes while exploiting linkage disequilibrium among nearby SNPs. We combined the reference individuals from Greenland (n = 181) and Europe (n = 8275), with the unrelated admixed Greenlanders (n = 1582) to construct the dataset for this analysis (n = 10038). First, we painted each reference individual using all other reference individuals, then, we painted each admixed Greenlander using all reference individuals. We specified constant mismatch (μ = 2.04 × 10e-5) and switch rate (*N*_e_ = 103.35) parameters across all analyses, which we estimated as the weighted mean values using data from chromosomes 1, 4, 15, and 22 in a subset of 168 individuals chosen to represent all reference populations, using 10 iterations of the expectation-maximization (EM) algorithm implemented in ChromoPainter. For all these analyses, we used the same recombination map as during haplotype phasing.

ChromoPainter quantifies coancestry using two different measures, one based on the length of the genome copied from each donor in centiMorgans (cM), deemed “chunk lengths” by the program, and the second based on simple counting of the number of distinct ancestry chunks copied from each donor, deemed “chunk counts.” Unless otherwise noted, we used the chunk lengths measure in downstream analyses. For summaries of the ChromoPainter analysis, see Table S1 and Figure S3.

#### Ancestry contributions from the European reference countries

We estimated the ancestry contributions from each European reference country and Greenland with SOURCEFIND (v2),^16^ based on summaries of the coancestry matrix estimated by ChromoPainter. We summarized the ChromoPainter output into a vector of length 15 for each admixed Greenlander and reference individual, with this vector containing the proportion of DNA by which that person is painted by individuals from each of the 15 reference populations. For each reference population, we averaged these vectors across individuals. We then applied SOURCEFIND to form the vector of each admixed Greenlander as a mixture of those from the reference populations. This is a Markov Chain Monte Carlo (MCMC) approach which puts a prior on the expected number of contributing reference populations and provides an estimate of the genetic contribution of the unadmixed Greenlandic Inuit and each of the 14 different European countries to the ancestry of the admixed individuals in Greenland, while accounting for sample size differences among reference populations. We conducted this analysis in two ways: 1) to each admixed Greenlander individually 2) to the entire set of admixed Greenlanders as a group. The individual-based analysis allowed us to investigate the range of individual-level patterns of European ancestry, while the group analysis considers a large number of individuals at once and estimates the ancestry sources of the mathematically-average admixed Greenlander. The later was done to reduce the noise from averaging the estimates of the individual-based analysis. For additional analyses related to validating the ancestry inference, please see Data S2.

To ensure convergence was reached in the SOURCEFIND analyses, we ran 5 MCMC chains for each analysis and compared variance within and between separate chains with the Rhat diagnostic.^36^ Each chain was run with 1M iterations, a 100K burn-in and a thinning factor of 1000. We tested that we discarded enough to burn-in by computing Rhat while discarding the first 500K iterations and compared these values to the shorter burn-in (data not shown). For each ancestry source in each individual the Rhat diagnostic was consistent with MCMC convergence; mean Rhat across all chains was 1.0001, and the max value was 1.0044. For most of our SOURCEFIND analyses we used default priors with eight eligible sources and a mean of four sources expected to contribute. However, we tested if the results were robust to choice of prior by also running additional analyses with a more sparse prior with eight eligible sources and two sources expected to contribute.

#### Investigating European admixture in the last few generations

To investigate the timing of European admixture, we assigned local ancestry, either Inuit or European, in each admixed Greenlandic individual using RFMix (v2).^27^ In this analysis, we used the same Inuit reference individuals as in the ChromoPainter analysis, along with CEU individuals from 1000G to represent the European ancestry, this allowed us to utilize the larger number of overlapping loci with the 1000G dataset. RFMix was run with default parameters, except we specified two different admixture dates, either 3 or 8 generations ago, to ensure that our results were robust to this choice. We used genotype data from the merged Greenlandic and 1000 Genomes datasets with 521,622 sites, split by chromosome and phased without a reference panel. After phasing, the reference Inuit and CEU individuals were used as the ancestry references for local ancestry inference in the admixed Greenlanders.

We summarized the results for each individual by calculating the fraction of the genome, in cM, that has either two Inuit alleles, two European alleles, or one Inuit and one European allele. We found a few chromosomal regions, such as near the edge of chromosomes, with local ancestry fractions that were outliers relative to the rest of the genome, suggesting potential problems with the inference of local ancestry in these regions, or local genomic factors affecting ancestry. To address this, we removed 88 out of 26008 (0.3%) genomic windows of local ancestry calls with less than 62.5% Inuit ancestry or with more than 72.5% Inuit ancestry, for a total exclusion of 3.76 cm.

## Supplementary Material

Supplementary 1

Supplementary 2

Supplementary 3

## Figures and Tables

**Table 1.** Inferred ancestry across 1,582 admixed Greenlandic individuals

	No. of reference individuals	Individual-based			Group-based
		≥1%	≥5%	≥20%	

Belgium	537	0.0% (0)	0.0% (0)	0.0% (0)	0.1%
Denmark	327	76.4% (1,208)	69.5% (1,100)	35.8% (567)	31.6%
Finland	580	0.6% (9)	0.3% (5)	0.1% (1)	0.0%
France	478	0.0% (0)	0.0% (0)	0.0% (0)	0.2%
Greenlandic Inuit	181	98.3% (1,555)	98.3% (1,555)	97.4% (1,541)	65.6%
Germany	1,000	0.1% (1)	0.1% (1)	0.0% (0)	0.3%
Ireland	344	0.2% (3)	0.2% (3)	0.0% (0)	0.1%
Italy	745	0.0% (0)	0.0% (0)	0.0% (0)	0.2%
The Netherlands	1,000	0.1% (1)	0.1% (1)	0.1% (1)	0.1%
Northern Ireland	61	0.0% (0)	0.0% (0)	0.0% (0)	0.1%
Norway	942	17.8% (281)	6.2% (98)	1.1% (18)	0.7%
Poland	57	0.1% (2)	0.1% (1)	0.0% (0)	0.2%
Spain	204	0.0% (0)	0.0% (0)	0.0% (0)	0.2%
Sweden	1,000	3.5% (56)	1.3% (20)	0.1% (1)	0.3%
UK	1,000	0.3% (5)	0.2% (3)	0.1% (1)	0.2%

Results from both individual-based and group-based analyses are shown. The first column gives the number of reference individuals from each source. For individual-based, the values shown are the assignment to country at 1%, 5%, and 20% ancestry thresholds. The percentage values are the percentages of admixed Greenlandic individuals inferred to have at least 1%, 5%, or 20% ancestry from each source country. The numbers in parentheses are the number of individuals in each category. To be counted here, an individual must have had at least 1%, 5%, or 20% ancestry with a posterior probability above 99%. For group-based, the values shown are the percentage of the ancestry of the group of 1,582 admixed Greenlanders inferred to come from each country. All the results were inferred using SOURCEFIND. See also Table S2.

**Table:** KEY RESOURCES TABLE

REAGENT or RESOURCE	SOURCE	IDENTIFIER

Biological samples		

European genotype data	^20,21^	EGAD00000000120, EGAD00010000124, EGAD00010000288, EGAS00001002641
Greenlandic genotype data (Metabochip)	^22^	EGAD00010001427, EGAD00010001428
1000 Genomes data	^23^	CHB, YRI, CEU

Deposited data		

Greenlandic genotype data (MEGA)	this paper, available at https://www.ebi.ac.uk/ega/studies/EGAS00001004933	EGAS00001004933

Software and algorithms		

CHROMOPAINTER (v2)	^15^	https://people.maths.bris.ac.uk/~madjl/finestructure-old/chromopainter_info.html
GLOBETROTTER (v Dec.30.2016)	^24^	https://people.maths.bris.ac.uk/~madjl/finestructure/globetrotter.html
fineSTRUCTURE (v2, v4)	^15^	https://people.maths.bris.ac.uk/~madjl/finestructure/finestructure.html
SOURCEFIND (v2)	^16^	Contact Garrett Hellenthal at ghellenthal@gmail.com
ADMIXTURE (v1.3.0)	^25^	http://dalexander.github.io/admixture/
PLINK (v1.9)	^26^	https://www.cog-genomics.org/plink2
RFMix (v2)	^27^	https://github.com/slowkoni/rfmix
relateAdmix	^28^	https://github.com/aalbrechtsen/relateAdmix
PCAngsd	^29^	https://github.com/Rosemeis/pcangsd

## Highlights

- The present-day Greenlandic population has substantial amounts of European ancestry
- Denmark is the main source of this European ancestry
- There is little evidence of European ancestry from pre-colonial European contact
- The timing of much of the European admixture is very recent
