# Supplementary material for: The genetic history of Greenlandic-European contact
Source: Curr Biol. Author manuscript; Available in PMC 2021 Jul 16. (PMC8284823; doi:10.1016/j.cub.2021.02.041)
Supplement: Supplementary 1 [file NIHMS1718974-supplement-Supplementary_1.pdf]

Current Biology, Volume *31*

Supplemental Information

The genetic history

of Greenlandic-European contact

Ryan K. Waples, Aviaja L. Hauptmann, Inge Seiding, Emil Jørsboe, Marit E. Jørgensen, Niels Grarup, Mette K. Andersen, Christina V.L. Larsen, Peter Bjerregaard, Garrett Hellenthal, Torben Hansen, Anders Albrechtsen, and Ida Moltke

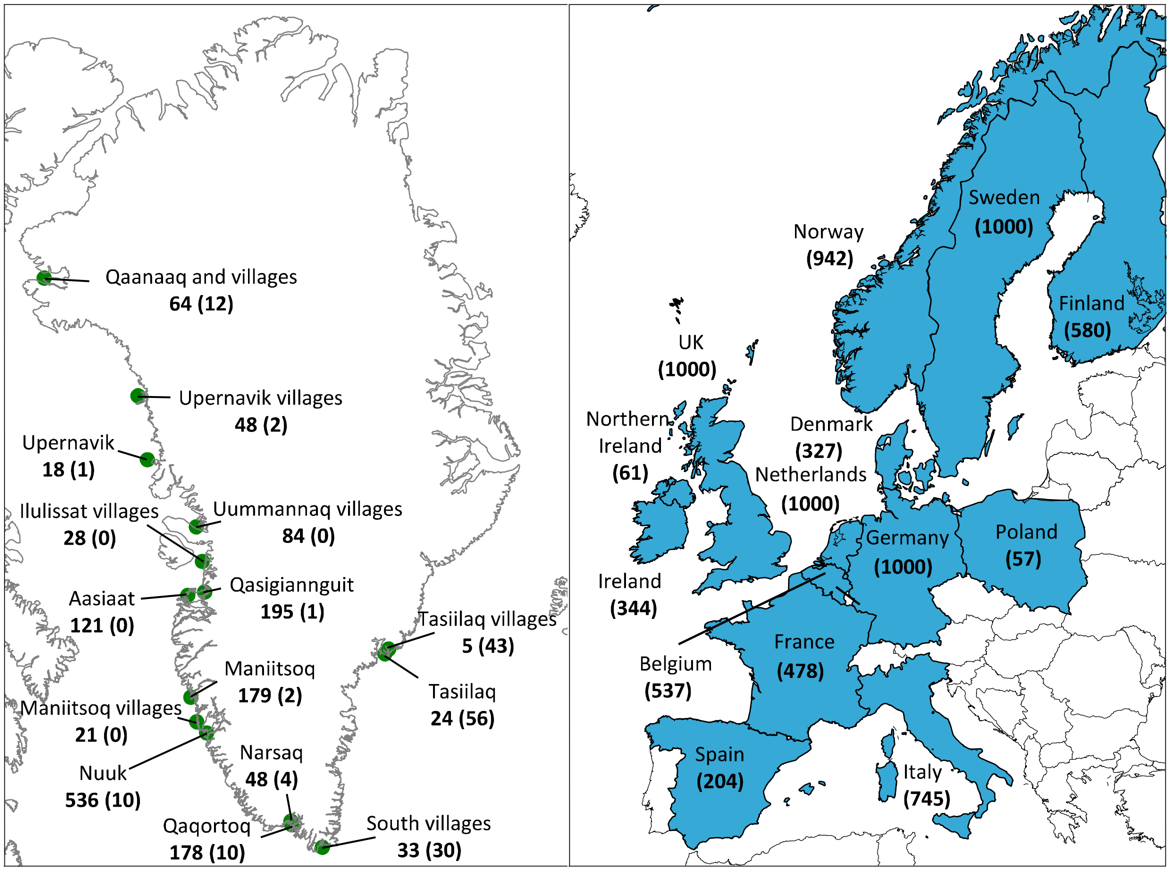

Figure S1. Geographic locations of Greenlandic and European individuals included in the study, related to STAR Methods. Numbers outside parentheses give the number of admixed Greenlandic individuals included from each location in Greenland. Numbers in parentheses give the number of individuals included in the reference panel from each location. There is a total of 1582 unrelated admixed Greenlanders and 8456 reference individuals.

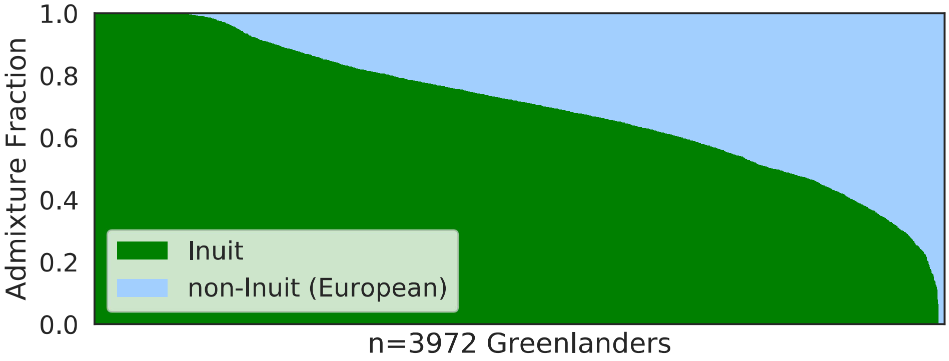

Figure S2. ADMIXTURE analysis (K=2), related to STAR Methods. Stacked bar plot of admixture proportions estimated for 3972 Greenlanders using an unsupervised ADMIXTURE analysis assuming two admixing populations (K=2), Inuit and European. Each individual is depicted as a thin vertical line with the Greenlandic Inuit ancestry proportion shaded green and the European ancestry proportion shaded light blue. This analysis was used to identify admixed and non-admixed Greenlanders for subsequent analyses.

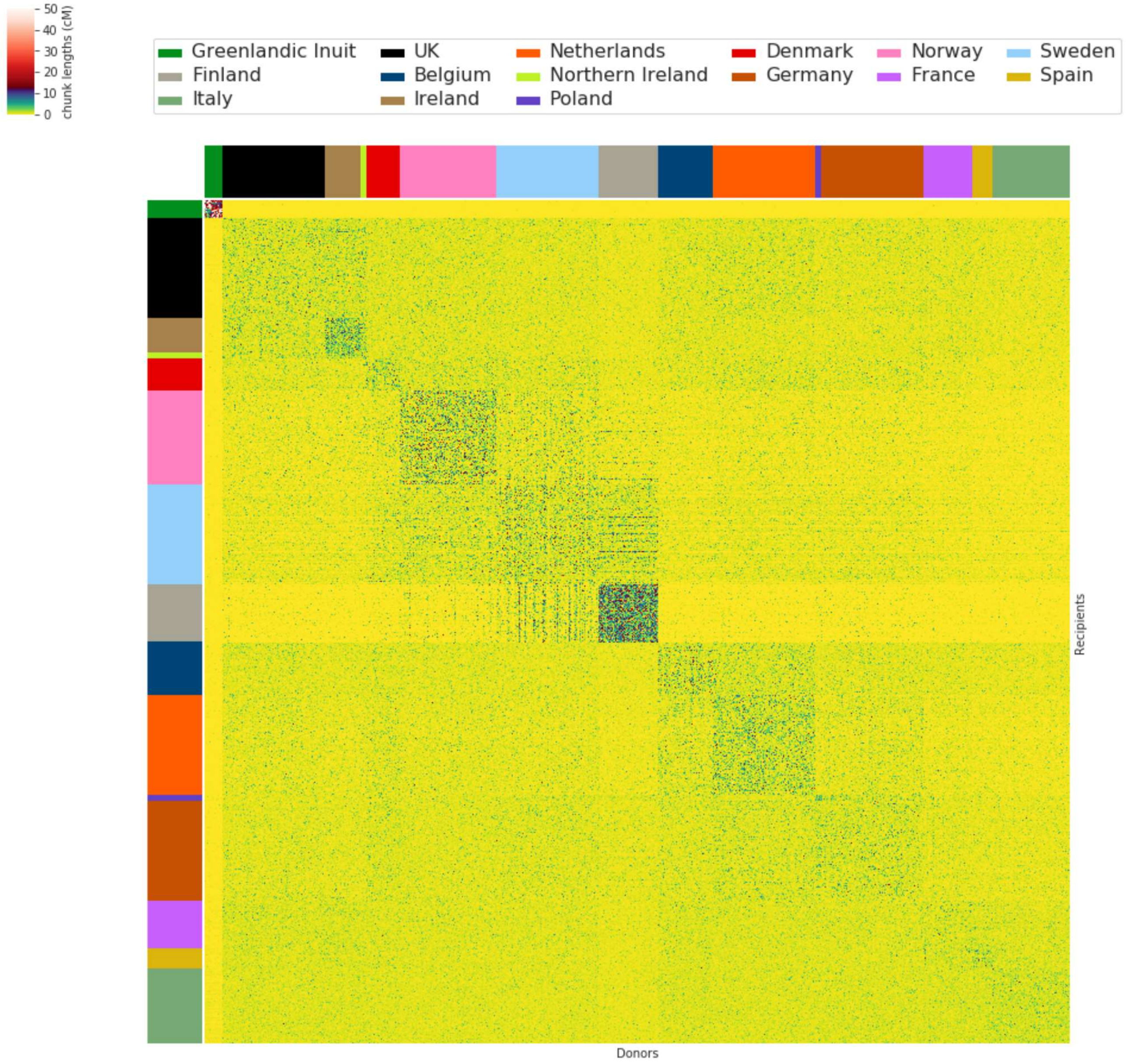

Figure S3. Heatmap of the coancestry matrix estimated by ChromoPainter, related to STAR Methods. The coancestry matrix was estimated between the 8456 reference individuals using ChromoPainter based on chunk lengths (the length of the genome copied from each donor in centiMorgans). Colors along the axes show the country of origin for each individual.

Chr	cM	# of SNPs	Mean # of chunks	Mean # of SNPs per chunk
1	292.1	10398	524.4	19.8
2	274.3	11109	502.4	22.1
3	227.1	9419	428.0	22.0
4	219.4	8233	402.4	20.5
5	208.6	7976	386.8	20.6
6	197.8	9336	367.4	25.4
7	189.5	7774	354.2	21.9
8	177.4	7189	312.7	23.0
9	179.4	6599	311.4	21.2
10	182.2	7070	331.7	21.3
11	161.5	6685	306.1	21.8
12	173.9	6697	320.3	20.9
13	128.3	4915	241.9	20.3
14	115.5	4430	224.2	19.8
15	150.8	4346	233.8	18.6
16	130.7	4496	246.4	18.2
17	127.9	4039	243.5	16.6
18	119.7	3907	224.5	17.4
19	106.6	2914	201.7	14.4
20	109.8	3698	207.8	17.8
21	63.5	2251	120.8	18.6
22	72.4	2221	133.7	16.6

Table S1. Chromosome-level summary of ChromoPainter analyses, related to STAR Methods. Summary of ChromoPainter results from each chromosome showing the number of SNPs and the number of distinct ancestry chunks on each chromosome. Chunk values are averages across all analyzed individuals, including both reference and admixed individuals.

With original prior	>=1% 95.0%	>=1% 99.0%	>=1% 99.9%	>=5% 95.0%	>=5% 99.0%	>=5% 99.9%	>=20% 95.0%	>=20% 99.0%	>=20% 99.9%
Belgium	0.1%	0.0%	0.0%	0.0%	0.0%	0.0%	0.0%	0.0%	0.0%
Denmark	81.4%	76.4%	70.1%	75.2%	69.5%	63.3%	40.4%	35.8%	30.5%
Netherlands	0.2%	0.1%	0.1%	0.1%	0.1%	0.1%	0.1%	0.1%	0.1%
Finland	0.7%	0.6%	0.4%	0.3%	0.3%	0.3%	0.1%	0.1%	0.1%
France	0.0%	0.0%	0.0%	0.0%	0.0%	0.0%	0.0%	0.0%	0.0%
Greenlandic Inuit	98.3%	98.3%	98.3%	98.3%	98.3%	98.3%	97.5%	97.4%	97.3%
Germany	0.6%	0.1%	0.0%	0.3%	0.1%	0.0%	0.0%	0.0%	0.0%
Ireland	0.4%	0.2%	0.2%	0.3%	0.2%	0.0%	0.0%	0.0%	0.0%
Italy	0.0%	0.0%	0.0%	0.0%	0.0%	0.0%	0.0%	0.0%	0.0%
Northern Ireland	0.1%	0.0%	0.0%	0.0%	0.0%	0.0%	0.0%	0.0%	0.0%
Norway	23.0%	17.8%	13.3%	8.4%	6.2%	4.7%	1.3%	1.1%	1.1%
Poland	0.5%	0.1%	0.0%	0.2%	0.1%	0.0%	0.0%	0.0%	0.0%
Spain	0.0%	0.0%	0.0%	0.0%	0.0%	0.0%	0.0%	0.0%	0.0%
Sweden	5.7%	3.5%	2.1%	2.0%	1.3%	0.8%	0.2%	0.1%	0.1%
UK	0.5%	0.3%	0.2%	0.3%	0.2%	0.2%	0.1%	0.1%	0.1%
**With sparse prior**									
Belgium	0.1%	0.0%	0.0%	0.0%	0.0%	0.0%	0.0%	0.0%	0.0%
Denmark	81.7%	77.0%	70.9%	76.1%	70.9%	64.7%	42.0%	36.9%	31.0%
Netherlands	0.2%	0.1%	0.1%	0.1%	0.1%	0.1%	0.1%	0.1%	0.1%
Finland	0.7%	0.6%	0.4%	0.3%	0.3%	0.3%	0.1%	0.1%	0.1%
France	0.0%	0.0%	0.0%	0.0%	0.0%	0.0%	0.0%	0.0%	0.0%
Greenlandic Inuit	98.3%	98.3%	98.3%	98.3%	98.3%	98.3%	97.5%	97.4%	97.3%
Germany	1.2%	0.1%	0.1%	0.6%	0.1%	0.0%	0.1%	0.0%	0.0%
Ireland	0.4%	0.2%	0.2%	0.3%	0.2%	0.0%	0.0%	0.0%	0.0%
Italy	0.0%	0.0%	0.0%	0.0%	0.0%	0.0%	0.0%	0.0%	0.0%
Northern Ireland	0.1%	0.0%	0.0%	0.0%	0.0%	0.0%	0.0%	0.0%	0.0%
Norway	22.6%	17.5%	13.0%	8.3%	6.1%	4.9%	1.3%	1.1%	1.1%
Poland	0.4%	0.1%	0.1%	0.2%	0.1%	0.0%	0.0%	0.0%	0.0%
Spain	0.0%	0.0%	0.0%	0.0%	0.0%	0.0%	0.0%	0.0%	0.0%
Sweden	5.9%	3.7%	2.3%	2.4%	1.3%	0.8%	0.2%	0.1%	0.1%
UK	0.5%	0.3%	0.2%	0.4%	0.3%	0.2%	0.1%	0.1%	0.1%

Table S2. Assignment to country at 1%, 5% and 20% ancestry thresholds across 1582 admixed Greenlanders, related to Table 1. The percentage values shown are the percentage of individuals inferred to have at least 1%, 5% or 20% ancestry from each source country, as in Table 1 in the main text. Sub-columns give percentages at different posterior probability thresholds: 95.0%, 99.0% and 99.9% (99.0% was used in Table 1). Top sub-table shows results with the original prior used to obtain Table 1, bottom sub-table shows results with a more sparse prior. The assignment analysis was conducted with SOURCEFIND using an individual-based approach.

	Recent admixture	Older admixture
Greenlandic Inuit	33.7%	71.6%
European (combined)	66.3%	28.4%
Denmark	98.4%	85.7%
Norway	0.5%	3.8%
UK	0.1%	0.9%
Sweden	0.1%	1.6%
Germany	0.1%	2.1%
France	0.1%	1.3%
Northern Ireland	0.1%	0.5%
Belgium	0.1%	0.6%
Netherlands	0.1%	0.3%
Spain	0.1%	0.8%
Ireland	0.1%	0.4%
Italy	0.1%	1.1%
Poland	0.1%	0.9%
Finland	0.0%	0.1%

Table S3. Ancestry estimates for groups of two groups of admixed Greenlanders, related to Figure 3. Ancestry estimates for groups of admixed Greenlanders with (n=250) and without (n=1332) a European-ancestry parent, here denoted "recent admixture" and "older admixture", respectively. The Greenlandic Inuit and combined European values are raw means, whereas the values for specific countries are the percentages of the European ancestry they constitute (they are divided by the value of "European (combined)". The European countries are listed in order of their mean value in the recent admixture column.

Supplemental References

S1 Alexander, D.H., Novembre, J., and Lange, K. (2009). Fast model-based estimation of ancestry in unrelated individuals. Genome Res. *19*, 1655–1664.

S2 Lawson, D.J., Hellenthal, G., Myers, S., and Falush, D. (2012). Inference of population structure using dense haplotype data. PLoS Genet. *8*, e1002453.

S3 Moltke, I., Fumagalli, M., Korneliussen, T.S., Crawford, J.E., Bjerregaard, P., Jørgensen, M.E., Grarup, N., Gulløv, H.C., Linneberg, A., Pedersen, O., et al. (2015). Uncovering the genetic history of the present-day Greenlandic population. Am. J. Hum. Genet. *96*, 54–69.

S4 Pedregosa, F., Varoquaux, G., Gramfort, A., Michel, V., Thirion, B., Grisel, O., Blondel, M., Prettenhofer, P., Weiss, R., Dubourg, V., et al. (2011). Scikit-learn: Machine learning in Python. the Journal of machine Learning research *12*, 2825–2830.

S5 Hellenthal, G., Busby, G.B.J., Band, G., Wilson, J.F., Capelli, C., Falush, D., and Myers, S. (2014). A genetic atlas of human admixture history. Science *343*, 747–751.

S6 Leslie, S., Winney, B., Hellenthal, G., Davison, D., Boumertit, A., Day, T., Hutnik, K., Royrvik, E.C., Cunliffe, B., Lawson, D.J., et al. (2015). The fine-scale genetic structure of the British population. Nature *519*, 309.

S7 Chacón-Duque, J.C., Adhikari, K., Fuentes-Guajardo, M., Mendoza-Revilla, J., Acuña-Alonzo, V., Barquera, R., Quinto-Sánchez, M., Gómez-Valdés, J., Martínez, P.E., Villamil-Ramírez, H., et al. (2018). Latin Americans show wide-spread Converso ancestry and imprint of local Native ancestry on physical appearance. Nat. Commun. *9*, 5388.

S8 Browning, B.L. and Browning, S.R. (2013). Improving the Accuracy and Efficiency of Identity-by-Descent Detection in Population Data. Genetics *194*, 459–471. URL `https://www.genetics.org/content/194/2/459`.

S9 Martin, A.R., Karczewski, K.J., Kerminen, S., Kurki, M.I., Sarin, A.P., Artomov, M., Eriksson, J.G., Esko, T., Genovese, G., Havulinna, A.S., et al. (2018). Haplotype sharing provides insights into fine-scale population history and disease in Finland. The American Journal of Human Genetics *102*, 760–775.

S10 Byrne, R.P., van Rheenen, W., van den Berg, L.H., Veldink, J.H., McLaughlin, R.L., and Consortium, P.M.A.G. (2020). Dutch population structure across space, time and GWAS design. Nature Communications *11*, 4556. URL `https://doi.org/10.1038/s41467-020-18418-4`.

S11 Frandsen, N., Gulløv, H., Heinrich, J., Jensen, E.L., Marquardt, O., Rud, S., Seiding, I., Toft, P.A., and Thuesen, S. (2017). Grønland – Den arktiske koloni. In Danmark og kolonierne, H.C. Gulløv, ed. (København: Gads Forlag), pp. 46–107.

S12 Ebenesersdóttir, S.S., Sandoval-Velasco, M., Gunnarsdóttir, E.D., Jagadeesan, A., Guðmundsdóttir, V.B., Thordardóttir, E.L., Einarsdóttir, M.S., Moore, K.H.S., Sigurðsson, Á., Magnúsdóttir, D.N., et al. (2018). Ancient genomes from Iceland reveal the making of a human population. Science *360*, 1028–1032. URL `https://science.sciencemag.org/content/360/6392/1028`.
