# Supplementary material for: The genetic history of Greenlandic-European contact
Source: Curr Biol. Author manuscript; Available in PMC 2021 Jul 16. (PMC8284823; doi:10.1016/j.cub.2021.02.041)
Supplement: Supplementary 2 [file NIHMS1718974-supplement-Supplementary_2.pdf]

Identification of admixed and unadmixed Greenlanders

The analyses of European ancestry in Greenlanders presented in this manuscript rely on unadmixed Greenlanders to serve a reference for the Greenlandic Inuit ancestry.

To establish and ensure that these individuals were unadmixed and were able to serve as references for the Greenlandic Inuit ancestry, we used the results from a K=2 analysis with ADMIXTURE [S1] to create two sets of Greenlandic individuals based on their estimated percentage of Greenlandic Inuit ancestry. Individuals with >99% Greenlandic Inuit ancestry were deemed "unadmixed" and were used as a references for the Greenlandic Inuit ancestry component. Individuals with <99% Inuit ancestry were deemed "admixed" and were the subjects of the analyses characterizing European ancestry.

To ensure the quality of the the set of unadmixed individuals we checked that thee admixture proportions that we based it on were consistent with those we could obtain from several other analyses. First, we conducted a supervised K=4 analysis with ADMIXTURE, after merging the genotype data from the Greenlanders with data from three 1000 Genomes populations, China (HAN), Nigeria (YRI), the US (CEU). This analysis inferred similar ancestry proportions across the Greenlandic individuals except for seventeen Greenlandic individuals with >5% African or >7% Asian ancestry, which were excluded. Second, we also compared the admixture proportions to the results obtained with the analyses we performed for other purposes using ChromoPainter [S2] and they were consistent with the admixture proportions of individuals in the ADMIXTURE analyses:

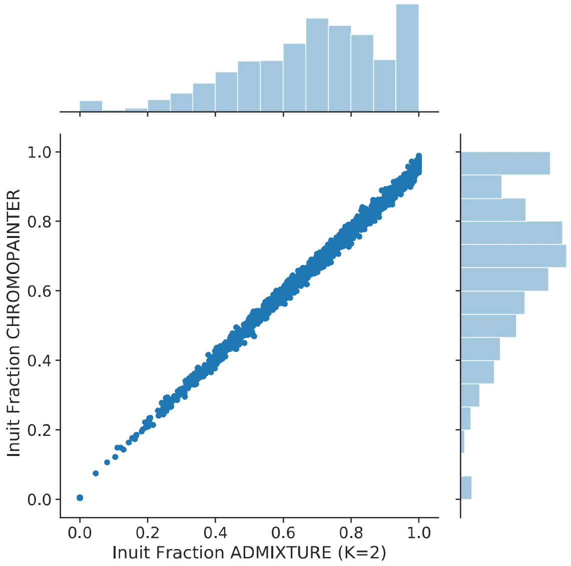

There are many reasons that we find it unlikely that all these analyses would miss-identify Greenlandic individuals with European admixture as "unadmixed". First, there is a large genetic difference between the Greenlandic Inuit and most other human populations, including European populations [S3], making it easier to differentiate these ancestries. Second, we have large sample sizes, with N=3972 Greenlanders. Third, we have reference individuals with European ancestry (e.g. 1000G CEU) that many other studies have established to be unadmixed. Fourth, we do find a range of admixture proportions across the Greenlandic individuals, with only some individuals being found as admixed. Taken together, this suggests that it is unlikely that the "unadmixed" individuals we used as a reference for the Greenlandic Inuit ancestry are actually admixed.
