# Supplementary material for: The genetic history of Greenlandic-European contact
Source: Curr Biol. Author manuscript; Available in PMC 2021 Jul 16. (PMC8284823; doi:10.1016/j.cub.2021.02.041)
Supplement: Supplementary 3 [file NIHMS1718974-supplement-Supplementary_3.pdf]

Further analyses to check the validity of results and study design

To investigate the validity of our results and the limitations our study design has potentially imposed on the conclusions that can be made from the results presented in this paper, we performed several additional analyses. These analyses included a principal component analysis (PCA), using an alternative method for ancestry assignment, a leave-one-out analysis, an evaluation of alternative groupings of reference individuals, and evaluating patterns of identity by descent (IBD). Below we will first describe the methods used for these analyses, then we will describe the results we obtained, and finally, based on these results and knowledge about the data set analysed, we will discuss whether our study design could have affected the conclusions of the study.

1 Methods

1.1 PCA of the ChromoPainter output

To assess the ChromoPainter results we visualized the chunk counts coancestry matrix with PCA, following the fineSTRUCTURE documentation [S2]. Specifically, we summed the painting vector from each individual over the donor groups (i.e. countries). This reduced the length 8456 painting vector representing copying from individuals to a length 15 copying vector representing copying from each country. We then combined these vectors into a matrix with a line for each individual and performed PCA on this matrix in Python using sklearn.decomposition.PCA [S4].

1.2 NNLS analysis

As an alternative to SOURCEFIND, we also ran the NNLS method, which is part of GLOBETROTTER [S5]. We ran it with prop.ind=1, props.cutoff=0.001, num.mixing.iterations=0 and applied it both to the data set analysed in the main text and to the reference samples alone in the leave-one-out setup described above.

1.3 Leave-one-out analysis of reference individuals

To assess the SOURCEFIND (and NNLS) results, we evaluated our ability to identify ancestry associated with each reference group with a leave-one-out procedure. We inferred the ancestry of each reference individual, while excluding them from the reference, in the same way we analysed each admixed Greenlander.

1.4 fineSTRUCTURE analyses

We performed unsupervised clustering of the reference individuals based on the Chromopainter output using the program fineSTRUCTURE (v0.0.5). We then summarized the results in a co-occurrence matrix representing how often each pair of individuals was placed into the same cluster. The co-occurrence matrix was calculated across nine independent runs, with 3M iterations each. The program was run with the following settings -c 0.18447, -I, with the scaling parameter c estimated as described in [S2]. For each run, the first 2.5M iterations were discarded as burn-in and the remaining iterations were sampled with a thinning ratio of 1000. The co-occurrence matrix contains, for each pair of individuals, the fraction of MCMC iterations that the pair was placed into the same cluster.

1.5 Re-analysis with ancestry groups obtained by hierarchical clustering.

To investigate if the results presented in the present study are robust to different ways of clustering the reference individuals, we hierarchically clustered the reference individuals using fineSTRUCTURE and repeated the main analyses with these clusters replacing countries as potential ancestry sources, similar to the approach taken in other studies [e.g. S6; S7]. Specifically, reference individuals were clustered with fineSTRUCTURE (v4.1.1) [S2] based on the chunkcounts matrix from ChromoPainter [S2]. Due to the large number of reference individuals (N=8456), we used the randomized greedy clustering method implemented in fineSTRUCTURE. We ran this greedy optimization 80 times and selected the run with the highest reported posterior. We then conducted hierarchical clustering ("tree-building") with fineSTRUCTURE, and cut the hierarchical clustering at three levels; K=20, 25, and 30 to produce K distinct clusters of the reference individuals. There is no obviously correct choice of K, and examining a range of values allowed us to investigate if the results were sensitive to this choice.

For each value of K, we used the clustering of reference individuals to establish potential ancestry sources for a separate set of SOURCEFIND [S7] analyses. These analyses were conducted in the same manner as the main analyses with: 2M iterations, a thinning interval of 10K, and an ancestry bin size of 0.5%. We ran both the group-based and individual-based analyses described in the main paper with these new references.

We summarized the results of these analyses in several ways. First we described the clusters and how they related to countries and thus the units used in the main analyses. Second, for the group-based analyses, we estimated the fraction of ancestry inferred from each cluster. For both the analyses, we labeled the clusters with a country label if >80% individuals in that cluster originated from a single country. We then summed the ancestry contributions from the clusters labeled with each country, and used these as proxies for ancestry from these countries. This labelling of clusters was meant to facilitate comparison to the country-based results presented in the main text. In addition to the >80% threshold, we also considered a >90% threshold to assign country labels, but found very similar results (results not shown).

1.6 IBD analysis

We inferred IBD segments between all pairs of individuals used in the ChromoPainter analysis (n=10038) with Refined-IBD (v17Jan20.102) [S8], using the same input data and recombination map as in the ChromoPainter analyses. Refined-IBD was run with default settings for windows size (40cM), minimum LOD score (3), minimum cM length (1.5 cM) and other parameters. We summarized IBD sharing between two countries as the mean IBD shared (in cM) across all pairs of individuals from the two countries and the IBD sharing within each country as the mean IBD shared (in cM) across all pairs of individuals from the country. In addition, we also considered only IBD segments with LOD>10, to focus on longer IBD segments representing more recent shared ancestry.

2 Results

We first performed two analyses to ensure that output from ChromoPainter, and thus the input to SOURCEFIND, actually allows the ancestry from the different countries to be distinguished: a principal component (PC) analysis of the ChromoPainter co-ancestry estimates and clustering analysis using fineSTRUCTURE [S2]. From the principal component (PC) analysis of the ChromoPainter results, we found that the first PC axis separates the reference Inuit from all the Europeans, with the admixed Greenlanders falling between the Europeans and the reference Inuit, and that subsequent axes

each tend to separate almost all the individuals from one or two European countries from the rest:

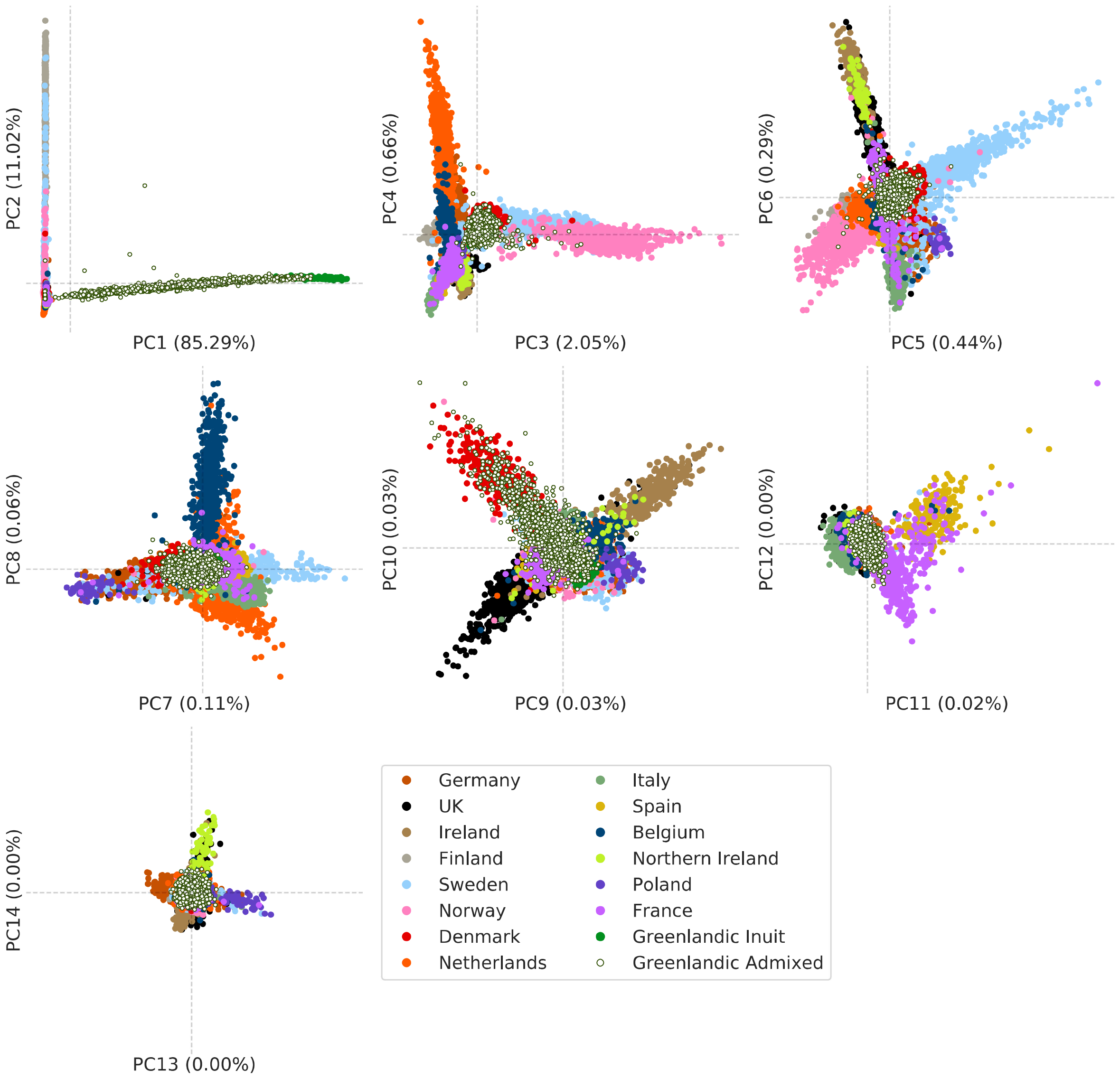

For example, the third PC axis separates most of the Norwegian individuals from those of the other countries, including Denmark, and the ninth and tenth PC axes combined separate the Danish individuals from most of the individuals from the remaining countries, suggesting that these data can indeed be used to distinguish ancestry from countries like Norway and Denmark that are genetically very similar. Notably, many admixed Greenlanders have a pronounced weighting on the ninth and tenth PC axes, projecting them coincident with the Danish individuals.

When using fineSTRUCTURE to genetically cluster the reference individuals based on the ChromoPainter output, we found that the reference individuals in general cluster according to country. This can be seen from the following heat map of the symmetric co-occurrence matrix for the reference individuals estimated with fineSTRUCTURE, where the shading of each cell indicates the posterior

probability of a pair of individuals being placed in the same cluster by the clustering procedure:

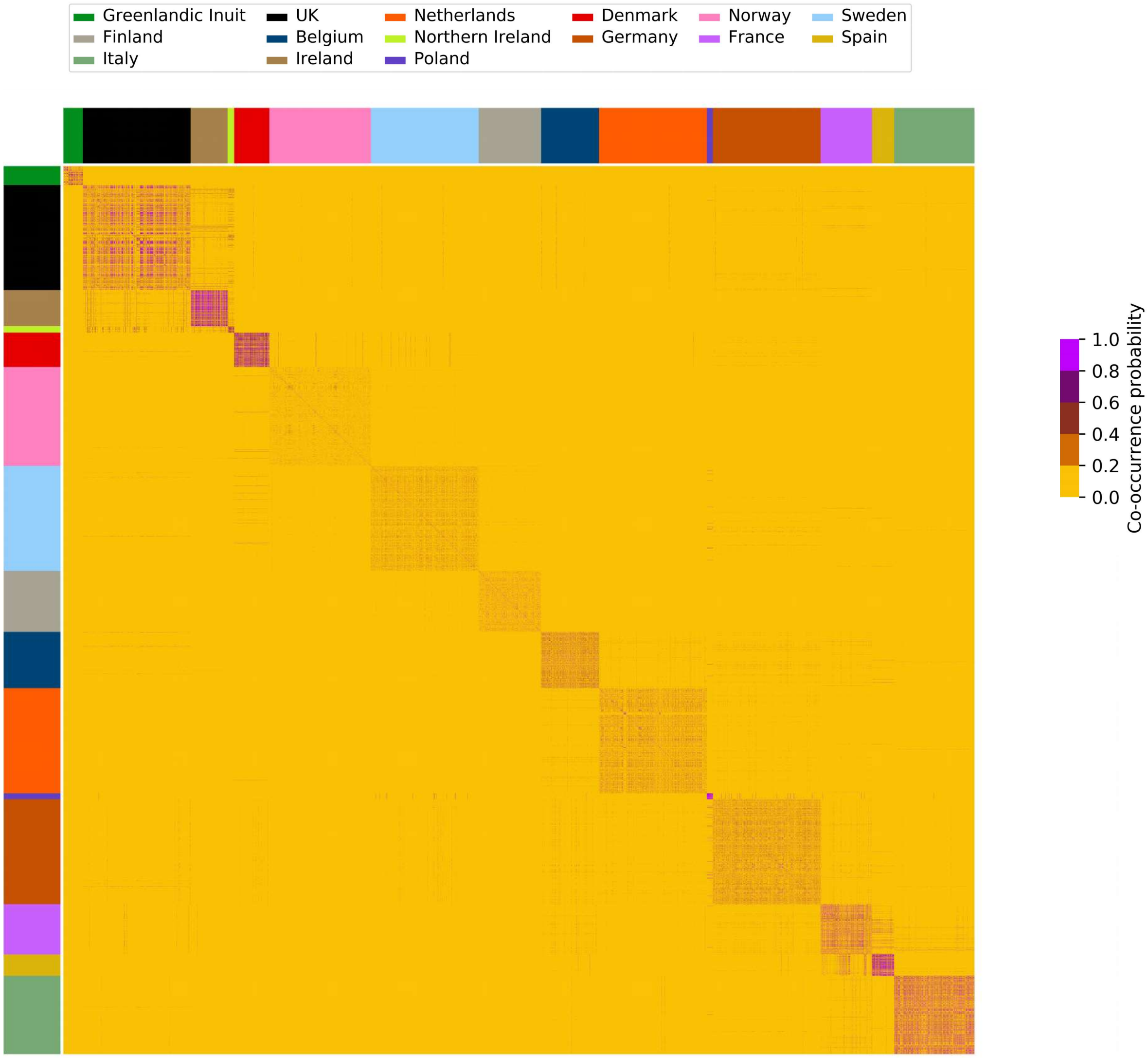

Hence it is indeed possible to distinguish between the different European countries based on the ChromoPainter results.

Second, we assessed the ability of SOURCEFIND to correctly assign ancestry to each source country using a leave-one-out strategy. This analysis demonstrated a high ability to identify the presence of ancestry from most reference countries, especially Denmark (97.9%), the Netherlands (99.0%) and Norway (98.7%), using a threshold of at least 5% ancestry with a posterior probability above 99% like in the analyses presented in the main text as can be seen from this table:

	Belgium	Denmark	Nether-lands	Finland	France	Green-landic Inuit	Germany	Ireland	Italy	Northern Ireland	Norway	Poland	Spain	Sweden	UK
							$\geq$ 5%								
Belgium	94.0%	0.0%	5.6%	0.2%	0.6%	0.0%	1.1%	0.2%	2.0%	0.2%	0.0%	1.1%	0.7%	0.0%	0.9%
Denmark	0.0%	97.9%	0.3%	0.3%	0.0%	0.0%	0.0%	0.0%	0.0%	0.0%	1.8%	2.8%	0.0%	3.1%	0.6%
Netherlands	13.1%	0.2%	99.0%	0.0%	0.0%	0.0%	0.2%	0.0%	0.9%	0.0%	0.3%	0.3%	0.3%	0.1%	0.1%
Finland	0.0%	0.0%	0.0%	100.0%	0.0%	0.0%	0.0%	0.0%	0.0%	0.0%	0.3%	0.9%	0.0%	10.9%	0.0%
France	3.8%	0.0%	0.4%	0.0%	63.2%	0.0%	0.0%	0.0%	6.7%	0.0%	0.0%	2.9%	16.7%	0.0%	0.0%
Greenlandic Inuit	0.0%	0.0%	0.0%	0.0%	0.0%	100.0%	0.0%	0.0%	0.0%	0.0%	0.0%	0.0%	0.0%	0.0%	0.0%
Germany	0.8%	2.0%	14.4%	0.1%	0.2%	0.0%	76.4%	0.2%	2.6%	0.0%	0.0%	22.1%	0.3%	0.3%	0.3%
Ireland	0.0%	0.0%	0.0%	0.0%	0.0%	0.0%	0.0%	93.9%	0.6%	3.5%	0.0%	0.0%	0.0%	0.0%	3.8%
Italy	0.3%	0.0%	0.0%	0.0%	0.1%	0.0%	0.0%	0.0%	98.9%	0.0%	0.0%	1.3%	0.1%	0.0%	0.3%
Northern Ireland	0.0%	0.0%	0.0%	0.0%	0.0%	0.0%	0.0%	19.7%	0.0%	85.2%	0.0%	0.0%	0.0%	0.0%	8.2%
Norway	0.0%	3.3%	0.1%	5.7%	0.0%	0.0%	0.2%	0.2%	0.1%	0.1%	98.7%	0.8%	0.0%	7.7%	0.3%
Poland	0.0%	0.0%	0.0%	0.0%	0.0%	0.0%	0.0%	0.0%	0.0%	0.0%	0.0%	100.0%	0.0%	0.0%	0.0%
Spain	0.0%	0.0%	0.0%	0.0%	0.0%	0.0%	0.0%	0.0%	1.0%	0.0%	0.0%	0.0%	98.5%	0.0%	0.0%
Sweden	0.2%	14.5%	0.5%	14.3%	0.0%	0.0%	1.4%	0.0%	2.6%	0.0%	16.1%	5.1%	0.4%	91.0%	0.4%
UK	0.2%	0.2%	0.3%	0.1%	0.0%	0.0%	0.4%	7.8%	0.5%	9.9%	0.8%	1.0%	0.0%	0.0%	86.2%

Furthermore, the rate of assignment of ancestry to non-origin countries was low, for example reference individuals from Denmark were assigned non-Danish ancestry from just three countries at rates above 1%: Norway (1.8%), Sweden (3.1%), and Poland (2.8%). And the results were similar using 1% or 20% ancestry thresholds:

	Belgium	Denmark	Nether-lands	Finland	France	Green-landic Inuit	Germany	Ireland	Italy	Northern Ireland	Norway	Poland	Spain	Sweden	UK
							$\geq$ 1%								
Belgium	94.4%	0.0%	6.7%	0.2%	0.6%	0.0%	1.1%	0.2%	2.0%	0.2%	0.0%	1.5%	0.7%	0.0%	1.1%
Denmark	0.0%	98.2%	0.9%	0.6%	0.0%	0.0%	0.0%	0.0%	0.3%	0.0%	4.0%	2.8%	0.0%	3.4%	0.6%
Netherlands	13.5%	0.3%	99.0%	0.0%	0.0%	0.1%	0.2%	0.0%	0.9%	0.0%	0.4%	0.3%	0.3%	0.1%	0.1%
Finland	0.0%	0.0%	0.0%	100.0%	0.0%	0.0%	0.0%	0.0%	0.0%	0.0%	1.2%	1.0%	0.0%	11.7%	0.0%
France	4.2%	0.0%	0.4%	0.0%	64.4%	0.2%	0.0%	0.2%	7.9%	0.0%	0.0%	2.9%	17.6%	0.0%	0.0%
Greenlandic Inuit	0.0%	0.0%	0.0%	0.0%	0.0%	100.0%	0.0%	0.0%	0.0%	0.0%	0.0%	0.0%	0.0%	0.0%	0.0%
Germany	0.9%	2.3%	15.7%	0.3%	0.3%	0.0%	77.5%	0.2%	3.1%	0.0%	0.0%	23.4%	0.3%	0.3%	0.3%
Ireland	0.0%	0.0%	0.3%	0.0%	0.0%	0.0%	0.0%	94.2%	0.6%	4.4%	0.0%	0.0%	0.0%	0.0%	4.4%
Italy	0.3%	0.0%	0.0%	0.0%	0.1%	0.0%	0.0%	0.0%	99.1%	0.0%	0.0%	1.3%	0.1%	0.0%	0.3%
Northern Ireland	0.0%	0.0%	0.0%	0.0%	0.0%	0.0%	0.0%	19.7%	0.0%	88.5%	0.0%	0.0%	0.0%	0.0%	8.2%
Norway	0.0%	3.7%	0.3%	8.9%	0.0%	0.1%	0.2%	0.2%	0.1%	0.1%	98.7%	0.8%	0.0%	9.1%	0.3%
Poland	0.0%	0.0%	0.0%	0.0%	0.0%	0.0%	0.0%	0.0%	0.0%	0.0%	0.0%	100.0%	0.0%	0.0%	0.0%
Spain	0.0%	0.0%	0.0%	0.0%	0.0%	0.0%	0.0%	0.0%	1.0%	0.0%	0.0%	0.0%	98.5%	0.0%	0.0%
Sweden	0.2%	16.3%	0.7%	18.1%	0.0%	0.1%	1.5%	0.0%	2.6%	0.0%	20.5%	5.5%	0.6%	91.3%	0.4%
UK	0.3%	0.3%	2.1%	0.3%	0.0%	0.0%	0.4%	8.3%	0.5%	10.6%	1.3%	1.1%	0.0%	0.0%	86.6%
							$\geq$ 20%								
Belgium	91.6%	0.0%	2.6%	0.0%	0.2%	0.0%	0.9%	0.2%	1.5%	0.2%	0.0%	1.1%	0.6%	0.0%	0.9%
Denmark	0.0%	97.9%	0.3%	0.3%	0.0%	0.0%	0.0%	0.0%	0.0%	0.0%	0.9%	1.2%	0.0%	0.3%	0.6%
Netherlands	7.5%	0.1%	97.7%	0.0%	0.0%	0.0%	0.1%	0.0%	0.6%	0.0%	0.0%	0.0%	0.1%	0.0%	0.1%
Finland	0.0%	0.0%	0.0%	99.8%	0.0%	0.0%	0.0%	0.0%	0.0%	0.0%	0.0%	0.0%	0.0%	4.1%	0.0%
France	0.6%	0.0%	0.2%	0.0%	59.6%	0.0%	0.0%	0.0%	4.8%	0.0%	0.0%	1.9%	13.6%	0.0%	0.0%
Greenlandic Inuit	0.0%	0.0%	0.0%	0.0%	0.0%	100.0%	0.0%	0.0%	0.0%	0.0%	0.0%	0.0%	0.0%	0.0%	0.0%
Germany	0.1%	0.7%	6.3%	0.0%	0.1%	0.0%	72.1%	0.1%	1.7%	0.0%	0.0%	13.3%	0.2%	0.1%	0.3%
Ireland	0.0%	0.0%	0.0%	0.0%	0.0%	0.0%	0.0%	92.7%	0.3%	2.3%	0.0%	0.0%	0.0%	0.0%	3.2%
Italy	0.3%	0.0%	0.0%	0.0%	0.0%	0.0%	0.0%	0.0%	98.8%	0.0%	0.0%	0.7%	0.1%	0.0%	0.3%
Northern Ireland	0.0%	0.0%	0.0%	0.0%	0.0%	0.0%	0.0%	14.8%	0.0%	82.0%	0.0%	0.0%	0.0%	0.0%	6.6%
Norway	0.0%	2.2%	0.0%	1.7%	0.0%	0.0%	0.0%	0.1%	0.0%	0.1%	98.6%	0.2%	0.0%	2.3%	0.2%
Poland	0.0%	0.0%	0.0%	0.0%	0.0%	0.0%	0.0%	0.0%	0.0%	0.0%	0.0%	100.0%	0.0%	0.0%	0.0%
Spain	0.0%	0.0%	0.0%	0.0%	0.0%	0.0%	0.0%	0.0%	0.0%	0.0%	0.0%	0.0%	97.1%	0.0%	0.0%
Sweden	0.0%	6.5%	0.2%	8.8%	0.0%	0.0%	1.0%	0.0%	2.2%	0.0%	5.9%	3.5%	0.3%	88.0%	0.3%
UK	0.1%	0.0%	0.0%	0.0%	0.0%	0.0%	0.4%	5.5%	0.3%	6.8%	0.0%	0.6%	0.0%	0.0%	84.6%

Next, we assessed the robustness of the SOURCEFIND results by using the non-negative least squares (NNLS) method implemented in GLOBETROTTER [S5] as an alternative to SOURCEFIND. This gave qualitatively similar results (compare to Table 1 in the main text):

**NNLS**	# of reference individuals	group-based ancestry percentage
Belgium	537	-
Denmark	327	27.9%
Finland	580	-
France	478	0.6%
Greenlandic Inuit	181	64.9%
Germany	1000	0.8%
Ireland	344	-
Italy	745	1.3%
Netherlands	1000	-
Northern Ireland	61	-
Norway	942	2.6%
Poland	57	0.7%
Spain	204	-
Sweden	1000	0.9%
UK	1000	0.2%

However, consistent with the conclusion of [S7], the leave-one-out analysis performed with NNLS showed a reduced ability to recover the country of origin of the reference individuals compared to SOURCEFIND based on mean ancestry assignment back to each reference country, as shown below:

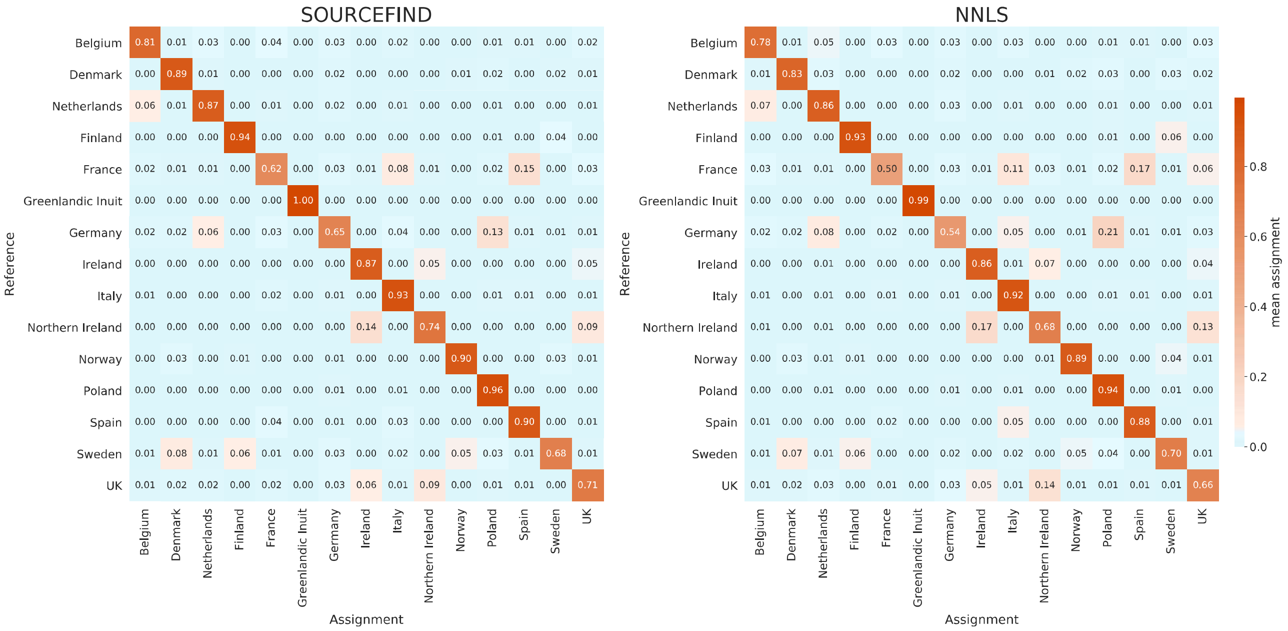

We also evaluated if our choice to use countries as the unit of analysis could lead to missing ancestry from genetically distinct groups not well represented by a country-of-origin clusters. To do so we repeated several of our analyses using groups based on an unsupervised hierarchical genetic clustering of the reference individuals as potential ancestry sources instead of groups based on country-of-origin.

This approach is similar to that taken by studies such as [S6] and [S7]. For the hierarchical genetic clustering we tried using three different numbers of clusters, K: 20, 25, and 30. Regardless of K the Greenlandic Inuit reference individuals (n=181) did not cluster with the European individuals, and formed two Greenlandic Inuit clusters, and the European individuals formed the remaining clusters, with sizes from n=74 to n=1019 at K=25. The European clusters were often composed of >80% of individuals from a single country, with 20 of 23 European clusters having >80% of individuals from a single country at K=25 as can be seen from the first two columns of this table:

Clusters for K=25	Country label (>80%)	Estimated % of the ancestry of the admixed Greenlanders from all clusters	Estimated % of the ancestry of the admixed Greenlanders from non-Greenlandic Inuit clusters
cluster 24	Greenlandic Inuit	49.51%	-
cluster 3	Denmark	31.99%	94.05%
cluster 25	Greenlandic Inuit	16.47%	-
cluster 16	Norway	0.23%	0.66%
cluster 19	Sweden	0.22%	0.64%
cluster 6	Germany	0.19%	0.55%
cluster 4	-	0.19%	0.55%
cluster 15	Norway	0.15%	0.45%
cluster 9	France	0.14%	0.40%
cluster 11	Italy	0.13%	0.38%
cluster 14	Norway	0.12%	0.35%
cluster 7	UK	0.12%	0.35%
cluster 13	Norway	0.11%	0.31%
cluster 10	-	0.09%	0.26%
cluster 20	Sweden	0.08%	0.25%
cluster 5	Belgium	0.06%	0.18%
cluster 1	Netherlands	0.04%	0.13%
cluster 8	Ireland	0.04%	0.12%
cluster 12	Norway	0.04%	0.11%
cluster 18	Sweden	0.03%	0.09%
cluster 2	Netherlands	0.02%	0.07%
cluster 17	-	0.02%	0.07%
cluster 22	Finland	0.01%	0.03%
cluster 23	Finland	0.00%	0.01%
cluster 21	Finland	0.00%	0.01%

The table also shows that when the clusters were labeled according to the countries with >80% of individuals from a single country, some European countries were represented by multiple clusters (e.g. Norway with five), some were represented by a single cluster (e.g. Denmark), while other countries were not represented by any cluster (e.g. Spain). The differences in the number of clusters per European country likely reflect the degree of genetic structure within each country, but may also be impacted by sample size differences between countries. We obtained similar results for the other two K values:

Clusters for K=20	Country label (>80%)	Estimated % of the ancestry of the admixed Greenlanders from all clusters	Estimated % of the ancestry of the admixed Greenlanders from non-Greenlandic Inuit clusters
cluster 19	Greenlandic Inuit	49.55%	-
cluster 2	Denmark	31.88%	93.74%
cluster 20	Greenlandic Inuit	16.44%	-
cluster 12	Norway	0.36%	1.06%
cluster 5	Germany	0.35%	1.03%
cluster 3	-	0.23%	0.69%

cluster 15	Sweden	0.20%	0.58%
cluster 11	Norway	0.17%	0.50%
cluster 6	UK	0.15%	0.45%
cluster 9	Italy	0.14%	0.41%
cluster 10	Norway	0.14%	0.40%
cluster 8	-	0.13%	0.39%
cluster 4	Belgium	0.07%	0.20%
cluster 7	Ireland	0.06%	0.18%
cluster 14	Sweden	0.05%	0.13%
cluster 1	Netherlands	0.04%	0.13%
cluster 13	-	0.03%	0.08%
cluster 17	Finland	0.01%	0.02%
cluster 18	Finland	0.00%	0.01%
cluster 16	Finland	0.00%	0.00%

Clusters for K=30	Country label (>80%)	Estimated % of the ancestry of the admixed Greenlanders from all clusters	Estimated % of the ancestry of the admixed Greenlanders from non-Greenlandic Inuit clusters
cluster 29	Greenlandic Inuit	49.63%	-
cluster 3	Denmark	31.99%	94.12%
cluster 30	Greenlandic Inuit	16.38%	-
cluster 20	Norway	0.19%	0.56%
cluster 23	Sweden	0.19%	0.55%
cluster 5	Germany	0.16%	0.47%
cluster 7	Germany	0.14%	0.41%
cluster 11	France	0.12%	0.37%
cluster 18	Norway	0.12%	0.34%
cluster 17	Norway	0.11%	0.33%
cluster 9	UK	0.10%	0.30%
cluster 19	Norway	0.10%	0.29%
cluster 14	Italy	0.09%	0.27%
cluster 13	-	0.08%	0.23%
cluster 24	Sweden	0.08%	0.23%
cluster 12	-	0.07%	0.22%
cluster 16	Norway	0.07%	0.21%
cluster 4	-	0.06%	0.19%
cluster 6	Belgium	0.06%	0.18%
cluster 8	-	0.06%	0.18%
cluster 10	Ireland	0.05%	0.14%
cluster 22	Sweden	0.03%	0.09%
cluster 2	Netherlands	0.03%	0.08%
cluster 15	Norway	0.03%	0.08%
cluster 1	Netherlands	0.03%	0.08%
cluster 21	-	0.02%	0.05%
cluster 26	Finland	0.00%	0.01%
cluster 27	Finland	0.00%	0.01%
cluster 25	Finland	0.00%	0.00%
cluster 28	Finland	0.00%	0.00%

Importantly, when using these clusters to perform group-based analyses, the two Greenlandic clusters combined consistently provided 66.0% of the ancestry of the admixed Greenlanders across all values of K (third column in the tables above). Furthermore, at K=25, Denmark was represented by a single cluster where >92% of individuals were from Denmark and this cluster was inferred to be the largest source of European ancestry in Greenland, with 94.05% of the European ancestry (see fourth column). No other country or cluster was inferred to contribute more than 1% of the European ancestry in Greenland, but clusters with with >80% of individuals from Norway and Sweden were the next two largest ancestry sources. These results are very consistent with the 91% Danish ancestry estimated in the group-based analyses in the main text. Again very similar results were found for K=20 and K=30. The results from the individual-based analyses using these clusters were also

consistent with the analyses presented in the main text. Specifically, the cluster with >92% of individuals from Denmark is inferred to be the largest source of European ancestry and clusters labeled with other countries showed similar inferred ancestry as in Figure 2 in the main text, with Norway the next largest source of ancestry, as can been seen from these figures:

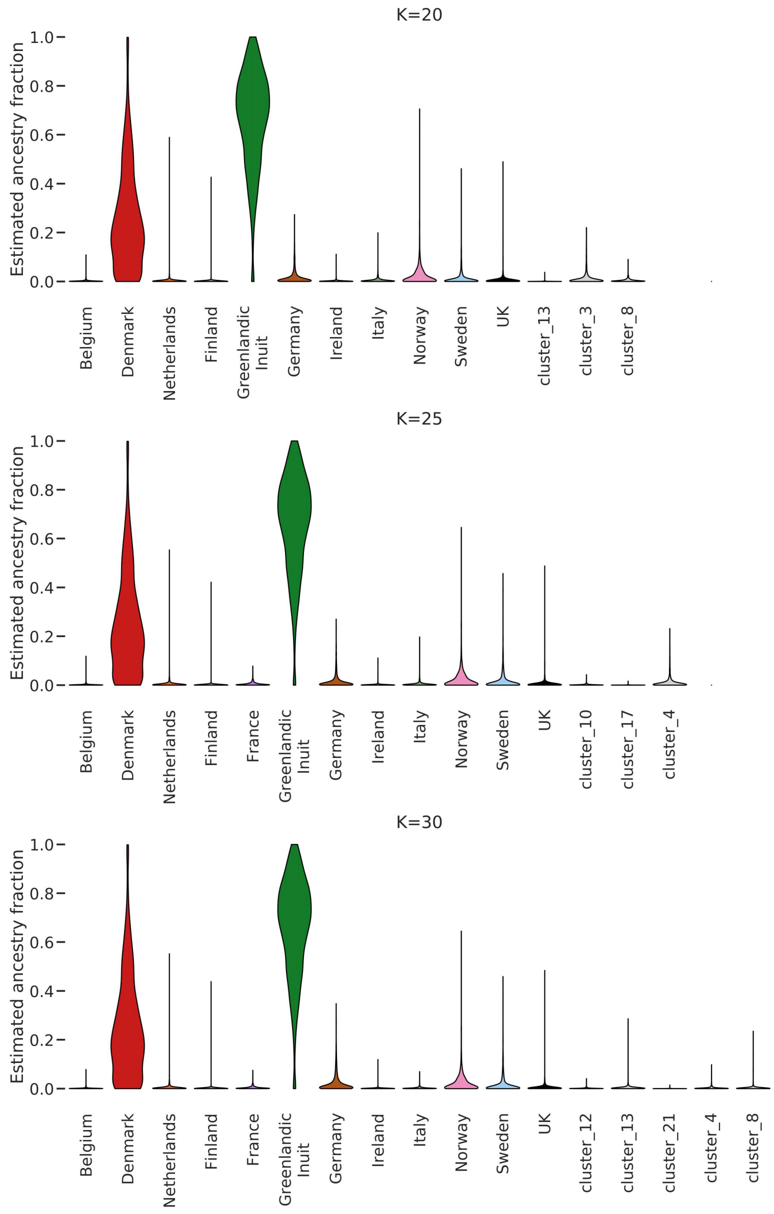

Hence all in all, this analysis led to very similar results to the country-of-origin based analyses.

Finally, we examined patterns of IBD sharing among the admixed Greenlanders and the reference individuals. We used ChromoPainter as a basis for all our analyses in the main text, because this method has been shown to be a particularly powerful method for answering the very fine scale ancestry questions posed in our study (REF). Hence this IBD analysis was not performed to get as fine-scaled answers, but merely to see if we get consistent results if we use another independent method. Specifically, we applied refinedIBD to the same data as ChromoPainter and inferred 221 million IBD

segments with an average segment length of 3.03 cM. When considering all IBD segments, mean IBD sharing was generally highest within a country, but not always, with the large and relatively diverse countries of France, Germany, Italy, and the UK not following this trend:

	Belgium	Den-mark	Nether-lands	Finland	France	Admixed Green-landers	Green-landic Inuit	Ger-many	Ireland	Italy	Nor-thern Ireland	Norway	Poland	Spain	Sweden	UK
All IBD segments																
Belgium	5.1421	4.8404	5.2427	3.7903	2.7821	1.6815	0.0345	3.9074	3.6508	1.5694	3.8581	4.5432	3.0967	2.0308	4.3197	4.0809
Denmark	4.8404	7.7790	6.3203	6.0209	3.1466	2.6446	0.0571	5.0290	4.2536	1.7695	4.5092	6.8379	4.3814	2.1438	6.5062	5.0121
Netherlands	5.2427	6.3203	8.2451	4.5222	3.1372	2.1808	0.0346	4.9629	4.2019	1.7351	4.5183	5.6938	3.5498	2.1579	5.4035	5.0671
Finland	3.7903	6.0209	4.5222	65.5594	2.6568	2.4587	0.5567	4.6917	3.3079	1.6097	3.5404	8.2338	6.4352	1.8165	13.6762	3.8311
France	2.7821	3.1466	3.1372	2.6568	2.5581	1.1326	0.0327	2.6742	3.1719	1.3623	3.1644	3.1399	2.4339	2.2114	2.9538	3.0660
Admixed Greenlanders	1.6815	2.6446	2.1808	2.4587	1.1326	283.6863	412.2828	1.7638	1.5174	0.6504	1.6460	2.5672	1.5772	0.7910	2.3838	1.7637
Greenlandic Inuit	0.0345	0.0571	0.0346	0.5567	0.0327	412.2828	858.2277	0.0451	0.0349	0.0373	0.0369	0.1631	0.0858	0.0387	0.1658	0.0362
Germany	3.9074	5.0290	4.9629	4.6917	2.6742	1.7638	0.0451	4.5867	3.4129	1.6066	3.6077	4.6355	5.2595	1.8796	4.7578	3.9250
Ireland	3.6508	4.2536	4.2019	3.3079	3.1719	1.5174	0.0349	3.4129	8.4198	1.6414	6.9038	4.6113	2.8799	2.3922	3.9390	5.1148
Italy	1.5694	1.7695	1.7351	1.6097	1.3623	0.6504	0.0373	1.6066	1.6414	1.1500	1.6494	1.7436	1.6444	1.1445	1.7006	1.6450
Northern Ireland	3.8581	4.5092	4.5183	3.5404	3.1644	1.6460	0.0369	3.6077	6.9038	1.6494	8.0093	4.7975	3.0568	2.4267	4.1974	5.2485
Norway	4.5432	6.8379	5.6938	8.2338	3.1399	2.5672	0.1631	4.6355	4.6113	1.7436	4.7975	11.5798	3.9976	2.1898	7.8140	4.9338
Poland	3.0967	4.3814	3.5498	6.4352	2.4339	1.5772	0.0858	5.2595	2.8799	1.6444	3.0568	3.9976	12.1505	1.6880	5.0370	3.0847
Spain	2.0308	2.1438	2.1579	1.8165	2.2114	0.7910	0.0387	1.8796	2.3922	1.1445	2.4267	2.1898	1.6880	3.1469	2.0384	2.2487
Sweden	4.3197	6.5062	5.4035	13.6762	2.9538	2.3838	0.1658	4.7578	3.9390	1.7006	4.1974	7.8140	5.0370	2.0384	9.0301	4.4885
UK	4.0809	5.0121	5.0671	3.8311	3.0660	1.7637	0.0362	3.9250	5.1148	1.6450	5.2485	4.9338	3.0847	2.2487	4.4885	5.0533

When only longer and more confidently called IBD regions were considered (LOD>10), the mean length was 6.5cM, and the pattern of excess IBD sharing within countries was even more evident:

	Belgium	Den-mark	Nether-lands	Finland	France	Admixed Green-landers	Green-landic Inuit	Ger-many	Ireland	Italy	Nor-thern Ireland	Norway	Poland	Spain	Sweden	UK
IBD segments with LOD >10																
Belgium	0.6080	0.1025	0.2345	0.0482	0.0470	0.0435	0.0005	0.0920	0.0569	0.0202	0.0675	0.0788	0.0512	0.0263	0.0711	0.0836
Denmark	0.1025	0.6776	0.1910	0.1554	0.0447	0.2228	0.0001	0.1247	0.0770	0.0206	0.0852	0.2644	0.1132	0.0231	0.2037	0.1120
Netherlands	0.2345	0.1910	0.8573	0.0652	0.0518	0.0788	0.0004	0.1621	0.0748	0.0226	0.0894	0.1239	0.0619	0.0243	0.1120	0.1282
Finland	0.0482	0.1554	0.0652	15.3059	0.0250	0.0833	0.0127	0.0845	0.0381	0.0157	0.0387	0.5950	0.1687	0.0129	1.7050	0.0500
France	0.0470	0.0447	0.0518	0.0250	0.0793	0.0199	0.0002	0.0399	0.0416	0.0190	0.0402	0.0403	0.0374	0.0700	0.0370	0.0466
Admixed Greenlanders	0.0435	0.2228	0.0788	0.0833	0.0199	79.6914	109.3774	0.0513	0.0337	0.0091	0.0390	0.1552	0.0440	0.0099	0.1005	0.0469
Greenlandic Inuit	0.0005	0.0001	0.0004	0.0127	0.0002	109.3774	360.5404	0.0006	0.0001	0.0001	0.0000	0.0108	0.0011	0.0001	0.0043	0.0003
Germany	0.0920	0.1247	0.1621	0.0845	0.0399	0.0513	0.0006	0.1831	0.0456	0.0245	0.0499	0.0835	0.2159	0.0222	0.0957	0.0746
Ireland	0.0569	0.0770	0.0748	0.0381	0.0416	0.0337	0.0001	0.0456	1.0457	0.0139	0.5711	0.1229	0.0216	0.0242	0.0682	0.2543
Italy	0.0202	0.0206	0.0226	0.0157	0.0190	0.0091	0.0001	0.0245	0.0139	0.0724	0.0139	0.0176	0.0296	0.0155	0.0192	0.0178
Northern Ireland	0.0675	0.0852	0.0894	0.0387	0.0402	0.0390	0.0000	0.0499	0.5711	0.0139	1.3050	0.1256	0.0348	0.0232	0.0741	0.3007
Norway	0.0788	0.2644	0.1239	0.5950	0.0403	0.1552	0.0108	0.0835	0.1229	0.0176	0.1256	1.9452	0.0677	0.0204	0.5097	0.1118
Poland	0.0512	0.1132	0.0619	0.1687	0.0374	0.0440	0.0011	0.2159	0.0216	0.0296	0.0348	0.0677	0.9307	0.0140	0.1275	0.0383
Spain	0.0263	0.0231	0.0243	0.0129	0.0700	0.0099	0.0001	0.0222	0.0242	0.0155	0.0232	0.0204	0.0140	0.2797	0.0192	0.0259
Sweden	0.0711	0.2037	0.1120	1.7050	0.0370	0.1005	0.0043	0.0957	0.0682	0.0192	0.0741	0.5097	0.1275	0.0192	0.9249	0.0818
UK	0.0836	0.1120	0.1282	0.0500	0.0466	0.0469	0.0003	0.0746	0.2543	0.0178	0.3007	0.1118	0.0383	0.0259	0.0818	0.2728

These results at least to some extent support the ChromoPainter based conclusions that countries are meaningful genetic units and that there is a potential to distinguish between different ancestries. Furthermore, of the Europeans, admixed Greenlanders as a group, shared the highest mean IBD with individuals from Denmark. This held for the default IBD analysis, as well as when selecting only the longer IBD segments. Mean IBD sharing between the admixed Greenlanders and other European countries was next highest with Norway and Sweden. There was no clear sign of Dutch or other sources of admixture that were missed in the ChromoPainter analysis. Hence the IBD analysis is consistent with the overall ChromoPainter based results.

3 Discussion

The study design used in this study consisted of three overall choices: the choice of programs used to analyse the data, the choice of how to apply these programs and finally the choice of dataset. We will discuss the potential impact of each these choices below based on the above described results and knowledge about the analysed dataset.

3.1 Choice of programs

We chose to use ChromoPainter and SOURCEFIND to perform our analyses with. This choice was based on prior papers showing that these methods are particularly powerful for resolving fine scale structure, e.g. [S7; S6; S9; S10], which was necessary given how genetically similar some of the possible European source populations are. This choice could in principle affect our results and make them invalid if the choice was not adequate. However, several additional analyses (PCA, fineStructure, and a leave-one-out analysis) suggest that the programs do indeed allow us to distinguish between ancestry from the different European countries. Also, when trying alternative similar methods like NNLS instead of SOURCEFIND we got similar results. Finally, even a very different approach based on IBD inference gave results that are consistent with the ChromoPainter/SOURCEFIND results, although these results are much harder to interpret. Hence, it seems that the results are fairly robust to our choice of methods.

3.2 How to apply these programs

The main issue to consider related to how the chosen programs were applied is the fact that we chose to use present-day countries as the unit of analysis, by describing each admixed Greenlander as a mixture of ancestries from different countries. This differs from the approach taken in many other similar analyses, where ancestry groups are based on an unsupervised genetic clustering of individuals. For example, [S7] defined 56 "surrogate" ancestry sources from across the world and used them to describe admixture in Latin Americans. We chose to use countries for two reasons. First, countries are easy to interpret and immediately recognisable. Second, the recent timescale of much of the gene flow into Greenland suggests that individuals from present-day European countries are likely to be reasonable proxies for the ancestry of individuals at the time of admixture. Importantly, the PCA of the co-ancestry matrix, the leave-one out analyses of the reference individuals, and fineSTRUCTURE analysis, all suggest this choice was reasonable. In particular, we demonstrated that we could distinguish ancestry from countries central to the main conclusions such as Denmark, Norway and the Netherlands and that using clusters obtained with unsupervised clustering instead of countries led to similar results.

3.3 Choice of data sets

The Greenlandic participants were sampled extensively from all regions of Greenland (Figure S1) and should therefore reflect the general genetic composition of the population well. A potential limitation is that none of the participants were sampled on Disko Island, the center of historical whaling activities in Greenland [S11]. However, according to self-reported birth location data, more than 50 of the participants were born on Disko Island (data not shown) suggesting that this region is actually well represented in our data set. There are also participants born in the regions in Greenland with German Moravian and Dano-Norwegian Lutheran missionaries. Hence the Greenlandic part of our dataset does not have any obvious limitations that would markedly affect the results. The reference data set from

Europe is also extensive, with individuals from almost all European countries with historical contact with Greenland (Figure 1), with a few notable exceptions. One of these is Portugal. However, we expect the lack of reference individuals from Portugal to have a minimal effect because we did include individuals from Spain, likely the closest match for Portuguese ancestry in our data, and we saw little Spanish ancestry in the admixed Greenlanders. Iceland and the Faroe Islands, two island nations whose history and location in the North Atlantic have facilitated contact with Greenland, were also not included due to a lack of publicly available genetic data. These two countries would potentially pose some difficulties to include, due to their recent history of substantial gene flow from source countries already included in this study (e.g., [S12]). However, this means that we do not know how large a part of the inferred Danish and Norwegian ancestry is also Icelandic or Faroese. Finally, the Frisians, an ethnic group that lives along the coastal parts of the Netherlands, Germany, and Denmark, is difficult to genetically distinguish as a separate ancestry source, as they likely represent some mixture of Germanic and Danish ancestries [S10]. However, if they indeed have such mixed ancestry, then the fact that we see very little Dutch and German ancestry suggests that the Frisians contributed very little, if any, to the genetic pool of the current Greenlandic population.
